# Spatial transcriptomic-metabolic features of tumor foci and tumor capsule in microvascular invasion with hepatocellular carcinoma: A spatial multi-omics study

**DOI:** 10.1371/journal.pmed.1004703

**Published:** 2026-05-15

**Authors:** Zhi-Hui Luo, Na Wang, Jingwei Zhao, Fei Long, Si Wu, Wei Zhong, Wei-Ming Chen, Bicheng Wang, Kun Wang, Yufeng Yuan, Jingjiao Zhou, Chunhui Yuan, Fubing Wang

**Affiliations:** 1 Department of Biology and Genetics, The College of Life Sciences and Health, Wuhan University of Science and Technology, Wuhan, Hubei, China; 2 Center for Single-Cell Omics and Tumor Liquid Biopsy, Zhongnan Hospital of Wuhan University, Wuhan, Hubei, China; 3 Department of Pathology, Renmin Hospital of Wuhan University, Wuhan, Hubei, China; 4 Department of Laboratory Medicine, Zhongnan Hospital of Wuhan University, Wuhan, Hubei, China; 5 Department of Pathology, Zhongnan Hospital of Wuhan University, Wuhan, Hubei, China; 6 Department of Laboratory Medicine, Hubei Cancer Hospital, Wuhan, Hubei, China; 7 Department of Hepatobiliary and Pancreatic Surgery, Zhongnan Hospital of Wuhan University, Wuhan, Hubei, China; 8 Department of Clinical Laboratory, Renmin Hospital of Wuhan University, Wuhan, Hubei, China; 9 Institute of Clinical Molecular Diagnosis, Wuhan University, Wuhan, Hubei, China; 10 Wuhan Research Center for Infectious Diseases and Cancer, Chinese Academy of Medical Sciences, Wuhan, Hubei, China; Peter MacCallum Cancer Centre, AUSTRALIA

## Abstract

**Background:**

Microvascular invasion (MVI) is closely related to the recurrence and metastasis of hepatocellular carcinoma (HCC), but the underlying cellular mechanism remains largely elusive. This study aims to elucidate the regional cellular discrepancy between MVI-positive (MVI^+^) and MVI-negative (MVI^−^) HCC by integrating Spatial transcriptomics (ST) and spatial metabolomics (SM).

**Methods and findings:**

ST and SM were performed on six tissue samples from four patients (including 2 MVI^+^, 2 MVI^−^, and 2 paratumor tissues), with the integration of 79 public single-cell RNA sequencing datasets of HCC. Patient identity was used as a covariate in the linear equation for regional differentially expressed gene analysis with the ST data. Clinical validation was conducted through multiplex immunofluorescence staining in 79 patients, together with external validation in the cancer genome atlas (TCGA)-liver hepatocellular carcinoma (LIHC) cohort (*n* = 299) and an independent microarray dataset (*n* = 62). For cell-type-specific metabolic profiling, spatial transcriptomic-metabolic registration was performed. The functional roles of key metabolites were further validated in vitro using inflammatory cancer-associated fibroblasts (iCAFs) derived from hepatic stellate cells (HSCs) and primary CAFs through co-culture models and various functional assays assessing cell proliferation, migration, and invasion. In the tumor lesion, a malignant *STMN1*^*+*^*HMGN2*^*+*^*GPC3*^*+*^ cell subtype enriched in MVI^+^ HCC was identified, which exhibited enhanced proliferative activity and was associated with poor prognosis. This finding was further confirmed in a local cohort of 79 patients, where multiplex immunofluorescence staining for the three genes (*STMN1*, *HMGN2*, and *GPC3*) showed significantly higher expression in the MVI^+^ group than in the MVI^−^ group (*p* = 0.046). Integrated SM analysis further revealed that this cell population underwent metabolic reprogramming characterized by suppressed glycerolipid metabolism. In the tumor capsule, iCAFs-related genes were downregulated in MVI^+^ cases, and iCAFs were located distally from the tumor boundary. Spatial metabolite mapping showed a strong correlation between taurine and iCAFs, and functional assays demonstrated that taurine promotes HCC proliferation and migration by suppressing iCAF activity. One limitation of this study is the small sample size of spatial omics data, which hinders a more complete molecular functional analysis of the *STMN1*^*+*^*HMGN2*^*+*^*GPC3*^*+*^ cell subtype and iCAFs in MVI^+^ HCC. Larger-scale ST cohorts are required to further validate and expand the findings of this study.

**Conclusions:**

This integrative spatial atlas proposes a hypothesis that there exists a highly proliferative and metabolically reprogrammed malignant cell subtype in the tumor lesion of MVI^+^ HCC, and that taurine in the tumor capsule modulates iCAF activity to influence tumor progression. The exploratory results provide mechanistic insights into MVI-related HCC progression and offer potential avenues for targeted therapeutic intervention of MVI^+^ HCC.

## Author summary

**Why was this study done?:**

- Microvascular invasion is a high tumor stage subtype of hepatocellular carcinoma (HCC) and is often correlated with tumor metastasis and recurrence.
- HCC has clear tumor foci and capsule areas (rich in immune cells and stromal cells) in the tissue region, but the differences in cellular functions between the microvascular invasion subtype and the common subtype in these tissue regions are not clear.
- This study aims to use spatial omics technologies to obtain the gene expression and metabolite distribution information in different tissue regions, and explore the changes in cells and their functions in the tumor area and capsule area of microvascular invasion-positive HCC.

**What did the researchers do and find?:**

- Using spatial transcriptomic and metabolomic data, combined with single-cell RNA sequencing, RNA sequencing gene expression data, and multiplex immunofluorescence staining, we identified and validated a specific subpopulation of *STMN1*^*+*^*HMGN2*^*+*^*GPC3*^*+*^ cancer cells within the microvascular invasion HCC tumor region. We found that this subpopulation exhibited enhanced proliferation and decreased glyceride metabolism.
- Using spatial omics data, RNA sequencing, and microarray data, we found the genes associated with inflammatory cancer-associated fibroblasts are downregulated in the microvascular invasion-positive HCC capsule. Spatial omics data demonstrated a positive correlation between the abundance of these cells and taurine concentration, while cell-based experiments confirmed that taurine can suppress the activity of these cells, thereby promoting cancer cell growth.

**What do these findings mean?:**

- *STMN1*^*+*^*HMGN2*^*+*^*GPC3*^*+*^ cancer cells may play a key role in microvascular invasion of HCC and may serve as potential targets for precision therapy.
- Inflammatory cancer-associated fibroblast (iCAF) cells may also serve as a potential therapeutic target for the microvascular invasive with HCC, and taurine may be a possible key intervention metabolite.
- Due to the small sample size and sample differences, the potential functional characteristics of *STMN1⁺HMGN2⁺GPC3⁺*  cancer cells and iCAFs may have not been fully detected.

## Abstract 3

Zhi-Hui Luo, Na Wang, Jingwei Zhao and colleagues use spatial transcriptomics and metabolomics to uncover regional cellular and metabolic differences in hepatocellular carcinoma associated with microvascular invasion, a key marker of aggressive disease.

## Introduction

Hepatocellular carcinoma (HCC) is the most prevalent primary liver tumor in the world, ranking sixth in global incidence and third in cancer-specific mortality [1]. The development of effective systemic therapies, such as liver transplantation, surgery, and immunotherapy, has substantially improved the outcomes for patients with HCC [2,3]. However, approximately 70% of the patients have disease recurrence within five years, which often results from intrahepatic metastasis [4]. Microvascular invasion (MVI), defined by neoplastic invasion of peritumoral microvasculature, is a critical determinant of its early recurrence and adverse survival outcomes [5,6]. By facilitating tumor dissemination via both intrahepatic and extrahepatic routes, MVI contributes to the initiation and progression of metastasis, resulting in low disease-free survival (DFS) rates in patients with MVI-positive (MVI^+^) HCC [7]. Notably, adjuvant hepatic arterial infusion chemotherapy with FOLFOX can significantly improve the DFS of patients with MVI^+^ HCC, underscoring the need for individualized therapeutic strategies specifically targeting MVI [8]. Therefore, elucidation of the intricate mechanisms underlying MVI can not only improve our understanding of HCC pathogenesis but also guide the development of potential targeted and precise interventions.

MVI represents a complex pathobiological phenomenon involving multifaceted cellular interactions and microenvironmental dynamics, particularly the reciprocal crosstalk between HCC cells and their stromal components [9]. Single-cell RNA sequencing (scRNA-seq) of MVI⁺ tumors has delineated distinctive molecular programs, which are characterized by dysregulated lipid metabolism, augmented angiogenic signaling, and increased proliferative capacity [7,9,10]. Notably, *TREM2 ⁺* macrophages are engaged in Midkine (*MDK*)-mediated intercellular communication with malignant cells, constituting a critical mechanism driving MVI development and tumor progression [10]. Furthermore, Apolipoprotein E positive (*APOE⁺*) macrophages and decorin (*DCN*)-expressing cancer-associated fibroblasts (CAFs) have been identified as another type of stromal contributors to MVI pathogenesis [7,11].

Spatial localization is a fundamental determinant of cellular functionality and interaction patterns, with distinct niche architectures demonstrating potent clinical relevance for patient stratification and therapeutic response prediction. For instance, Chen and colleagues identified an immune niche, which consists of antitumoral macrophages, CD8⁺ T cells, and natural killer (NK) T cells and is correlated with immunotherapy outcomes in small cell lung cancer [12], while C-X-C Motif Chemokine Ligand 14 positive (*CXCL14⁺*) CAFs at tumor-stroma boundary facilitate immune exclusion through extracellular matrix remodeling that impedes T-cell infiltration [13]. However, the spatial architecture of cellular ecosystems and molecular features that drive MVI formation remain poorly characterized at spatial resolution in HCC.

This study is hypothesis-generating and exploratory, focusing on spatial multi-omics data of MVI^+^ HCC. Our hypothesis was that MVI^+^ and MVI-negative (MVI^−^) HCC exhibit distinct cellular functional differences in both the tumor core region and the capsular region. These differences may be associated with specific cell types, as well as spatial distribution patterns of cells. Accordingly, this study has two main objectives: The primary objective was to identify whether there exist malignant cell populations in the tumor core of MVI^+^ HCC that are distinct from those in MVI^−^ HCC, and to characterize their major transcriptional and metabolic features. The second objective was to investigate whether functionally altered cell populations exist in the tumor capsule region of MVI^+^ HCC, and whether such functional changes are associated with specific metabolites.

## Methods

### Human HCC samples collection

Six independent HCC surgically resected specimens (including 2 MVI^+^ tumor boundary tissues, 2 MVI^−^ tumor boundary tissues, and 2 paratumor tissues) were collected from four patients at Zhongnan Hospital of Wuhan University for spatial transcriptomics (ST) and spatial metabolomics (SM) analyses. In addition, 79 formalin-fixed, paraffin-embedded (FFPE) HCC samples (21 MVI^+^, 58 MVI^−^) from the same hospital were obtained for multiplex immunofluorescence (MIF) validation.

### Ethics statement

HCC samples were collected in accordance with a protocol approved by the Research Ethics Committee of the Zhongnan Hospital of Wuhan University (2023073K). All patients gave written informed consent prior to participation. All diagnoses were histologically verified by a board-certified pathologist.

### 10× CytAssist ST sequencing

Freshly collected HCC and distant adjacent tissues were embedded in OCT and snap-frozen on dry ice to preserve their integrity. The tissues were sliced into 10 µm thick sections and then placed in the 10× Visium spatial slides (6.5 × 6.5 mm) capture area. Each capture area possessed 5,000 spots, with a diameter of approximately 55 µm. Tissue sections were subjected to methanol fixation, H&E staining, imaging, and destaining, following the 10× Genomics recommended procedure (CG000614).

Following the 10× Genomics experimental workflow (CG000495), probe hybridization and probe release were performed. Briefly, fresh-frozen liver sections were first mounted onto blank slides, followed by the addition of a predefined array of capture probes, each with a unique spatial barcode to overlay the tissue. Following tissue permeabilization and hybridization, mRNA molecules from the tissue were captured in situ by these probes. Subsequently, reverse transcription and amplification steps were performed to generate cDNA libraries, which were then spatially indexed based on the corresponding barcodes. The probes were then transferred to the 10× Visium CytAssist slide, and library construction was carried out using the Visium CytAssist Spatial Gene Expression for FFPE kit (PN-1000520). The resulting DNA libraries were subjected to high-throughput sequencing on Illumina NovaSeq.

### ST data processing

The fastq reads of ST data were mapped to GRCh38 by Space Ranger (https://www.10xgenomics.com/support/software/space-ranger/latest). Then, the gene expression matrix, cell barcode, cell spatial locations and corresponding H&E images were obtained. The expression data were normalized by the SCTransform [14], and integrated by SCTIntegration function in Seurat [15]. Then, principal component analysis (PCA) was performed by RunPCA function. The first 30 principal components were used in Findclusters function (shared nearest neighbor clustering) with resolution parameter ranging from 0.1 to 1. Considering both the accuracy and redundancy of clustering, we chose resolution 0.4 as the final result. Finally, 13 clusters were obtained for the integrated ST samples.

ST clusters were firstly manually annotated by the marker genes of hepatocyte genes *HAMP, MT1X,* and *MT1G* [16], immune/stromal genes of *CD3D*, *IGHG1*, *COL1A1,* and *ACTA2*, and malignant cell genes of *LAMB3, SERPINA12,* and *TUBB* [17–19]. Then, the annotation of clusters was refined by the cell deconvolution results and copy number variations (CNV).

### Spatial co-localization analysis

The cell percentages identified through deconvolution analysis were then used in the spatial co-localization analysis by mistyR [20]. Specifically, the cell percentage was split into six independent subsets based on the samples. Then they were built with an internal view model. Finally, the standardized importance median of all samples was summarized and defined as the cell type dependency relationship in different spatial contexts. Based on the internal view, a heatmap of cell type interactions was drawn with cutoff = 0.

### Differentially expressed genes analysis of ST

According to cluster annotation, we separated tumor region into four sections: Tumor (T) region (cluster ST_C1, ST_C6, ST_C8, ST_C10, ST_C12, ST_C13), tumor capsule (TC) region (cluster ST_C3 and ST_C5), paratumoral (PT) region (cluster ST_C2, ST_C4, ST_C9 and ST_C11), and transition state (TR) region (cluster ST_C7). Cluster ST_C4 in patient P1 possessed CNV scores and was located inside the cancer lesion according to H&E staining images. Therefore, it was classified as the T region.

Differentially expressed genes (DEGs) analysis was performed using the devil R-package [21] which applies a Bayesian Gamma–Poisson framework to restore patient-level independence and ensure statistically rigorous testing. For this analysis, spots from the T/TC region were considered. Raw counts were modeled with the fit_devil function, specifying MVI status (MVI^+^ or MVI^−^) as the main grouping variable in the design matrix. To address the issue of pseudo-replication inherent in spot-level analyses, patient identity was explicitly incorporated as the unit of replication by passing it to the test_de function through the “clusters” parameter. Differential expression between the MVI^+^ and MVI^−^ groups was then assessed with test_de (setting max_lfc = 50), and statistical significance was adjusted using the Benjamini–Hochberg procedure. Genes were considered significantly differentially expressed if they met the criteria of adjusted *P* value < 0.05 and |log2 fold change| > 1. Kyoto Encyclopedia of Genes and Genomes (KEGG) pathways were enriched for DEGs through clusterProfiler [22].

### Triple-positive gene selection

Firstly, we performed DEGs analysis between cluster SC_C6/SC_C7 and SC_C0/SC_C3/SC_C4/SC_C15 using Seurat. Among the top 30 DEGs, we selected the genes with high pct.1 (> 0.8). Then, we obtained *STMN1*, *TUBA1B*, *H2AFZ*, *HMGN2*, and *TUBB* as the dominant highly expression genes in cluster SC_6/SC_7. Secondly, we used the difference between pct.1 and pct.2 to measure the gene specificity in cluster SC_C6/SC_C7. We found that *STMN1* was the gene with the highest specificity in cluster SC_C6/SC_C7 (S7 Table). Thirdly, we used the Gepia2 website (http://gepia2.cancer-pku.cn/#index) to search for genes with similar expression patterns to *STMN1* in the cancer genome atlas (TCGA)-liver hepatocellular carcinoma (LIHC) data and found that *HMGN2* ranked second in correlation with *STMN1* among all genes. Therefore, we selected *HMGN2* as an auxiliary gene of *STMN1* to help us distinguish cluster SC_C6/SC_C7 from other subclusters. Fourth, considering that *STMN1* and *HGMN2* reflect proliferative and chromatin-remodeling programs that may also occur in non-malignant proliferative cells [23]. We incorporated *GPC3* to enhance hepatocellular carcinoma specificity. *GPC3* is a well-established HCC marker with minimal expression in non-tumor cells (Fig 5c). The final marker panel was therefore defined as *STMN1⁺/HMGN2⁺/GPC3⁺* cells, integrating data-driven discovery (*STMN1*, *HMGN2*) with a priori tumor-specific knowledge (*GPC3*).

**Fig 1:**
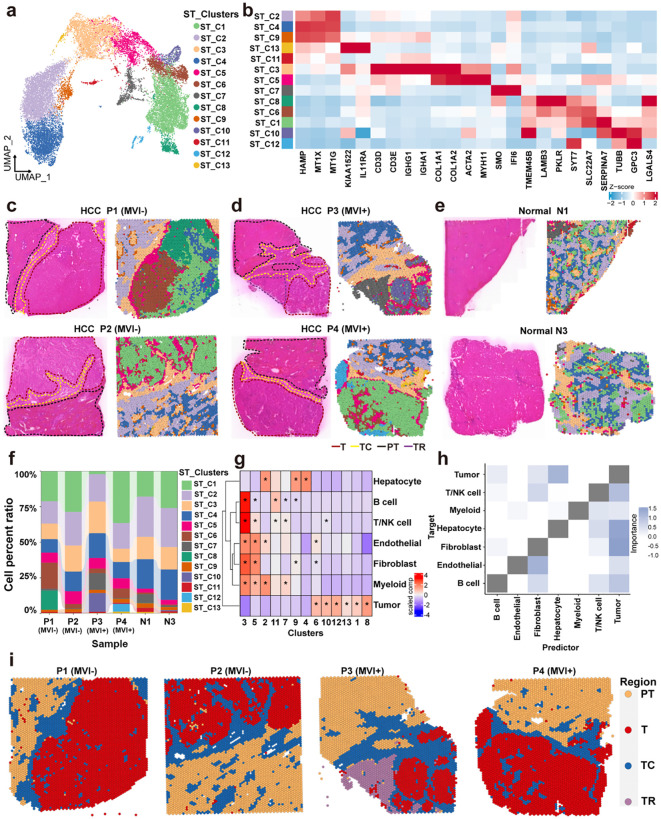
Regional delineation by cellular distribution in spatial transcriptomic samples. a, Uniform Manifold Approximation and Projection (UMAP) visualization of spot clusters for integrated samples. b, Heatmap showing the average expression of cell markers in spot clusters. c–e, Overview of the spatial transcriptomic spot clusters for MVI^−^ (c), MVI+ (d), and normal tissues (e). f, Stacked plot showing the proportion of each cluster in all samples. g, Scaled median cell-type compositions within each cluster. “*” represents a higher proportion of one cell type in a cluster compared with other clusters (Wilcoxon rank-sum test, *p.adjust* <0.05). Please see S2 Table for the exact *P* values corresponding to the asterisks “*”. **h**, Importance of the abundance of cell types in predicting other cell types within the same spot. **i**, Overview of regional delineation for T, PT, TC, and TR regions. MVI^+^, microvascular invasion positive; MVI^−^, microvascular invasion negative; ST, spatial transcriptomic; T, tumor; PT, paratumor; TC, tumor capsule; TR, transition state. NK, natural killer; HCC, hepatocellular carcinoma.

**Fig 2:**
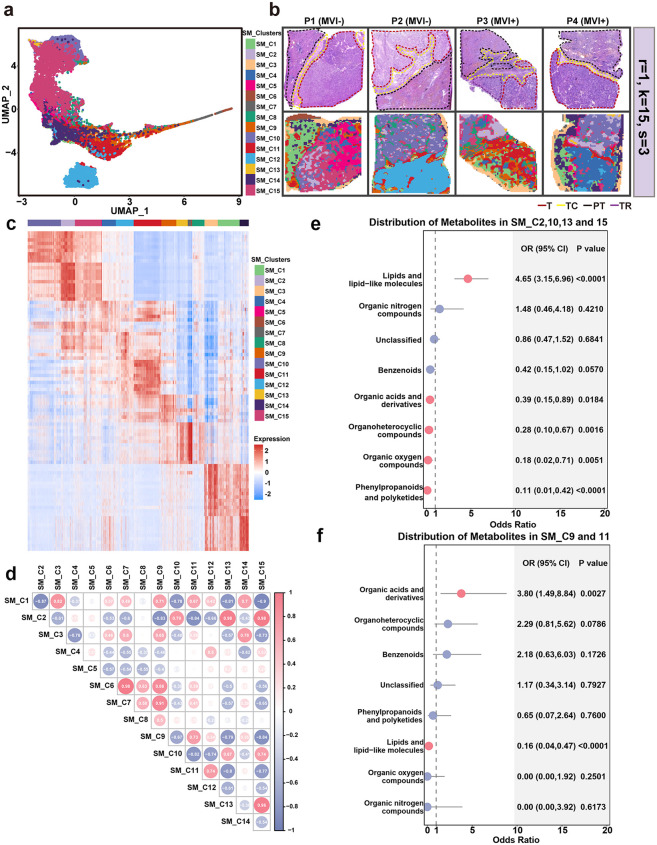
Regional accumulation of metabolites in MVI+ and MVI− HCC. a, UMAP visualization of clusters in SM data for all six samples. UMAP: Uniform manifold approximation and projection. **b**, Overview of spatial metabolite clusters in each sample. r denotes the smoothing radius, k represents the initial number of clusters, and s indicates the sparsity parameter used in spatial shrunken centroids clustering. **c**, Heatmap plot of top 10 marker metabolites in each cluster. The top 10 marker metabolites of each cluster were listed in S4 Table. **d**, Pearson correlation of metabolites in each cluster. **e and f**, Odds ratio of metabolite counts in the T region of clusters SM_C2, SM_C10, SM_C13 and SM_C15 (**e**) and clusters SM_C9 and SM_C11 (**f**) over that in the whole tissue by super class. Both MVI^+^ and MVI^−^ samples were included in the analysis. Red dots represent a *P-value* of Fisher’s test < 0.05, while blue dots represent a *P-value* of Fisher’s test > 0.05. MVI^+^, microvascular invasion positive; MVI^−^, microvascular invasion negative; SM, spatial metabolomics; T, tumor region; PT, paratumor region; TC, tumor capsule region; TR, transition state region; OR, odds ratio; CI, confidence interval.

### HCC tumor boundary and boundary distance

For the ST data, each spot was spatially surrounded by six spots. For the spots inside the cancer lesion, the annotations of the six surrounding spots also belong to the TC area. For the spots at the boundary of the cancer lesion, at least one of the six spots around them did not belong to the TC area. We divided the spots in the TC area into tumor core spots and tumor boundary spots according to this rule.

For non-tumor spots, we calculated the Euclidean distance between the spot and each boundary spot, and defined the shortest distance as the spatial distance from the spot to the boundary. This distance was then used in correlation calculations with the gene set scores.

### Gene signature score in ST data and TCGA RNA-Seq

Diacylglycerol biosynthesis process and the triglyceride biosynthetic process gene sets were downloaded from Gene Ontology (GO) database (https://www.geneontology.org/). CAFs gene signatures were derived from previous studies [24], and all significant genes (log2 fold change >0.25 and *P* value <0.05) for vascular CAFs (vCAFs), matrix CAFs (mCAFs), inflammatory CAFs (iCAFs), and antigen-presenting CAFs (apCAFs) were selected. For the ST data, these gene signatures were scored using the AddModuleScore function in the Seurat. LIHC bulk RNA-Seq data were downloaded from UCSC Xena. By matching with clinical data, they were classified in MVI^+^ and MVI^−^ cancer tissues. For these RNA-Seq data, the gene signatures were scored using the Gene Set Variation Analysis (GSVA) R package.

### Identification of MVI^+^ associated cancer cell populations

**Fig 3:**
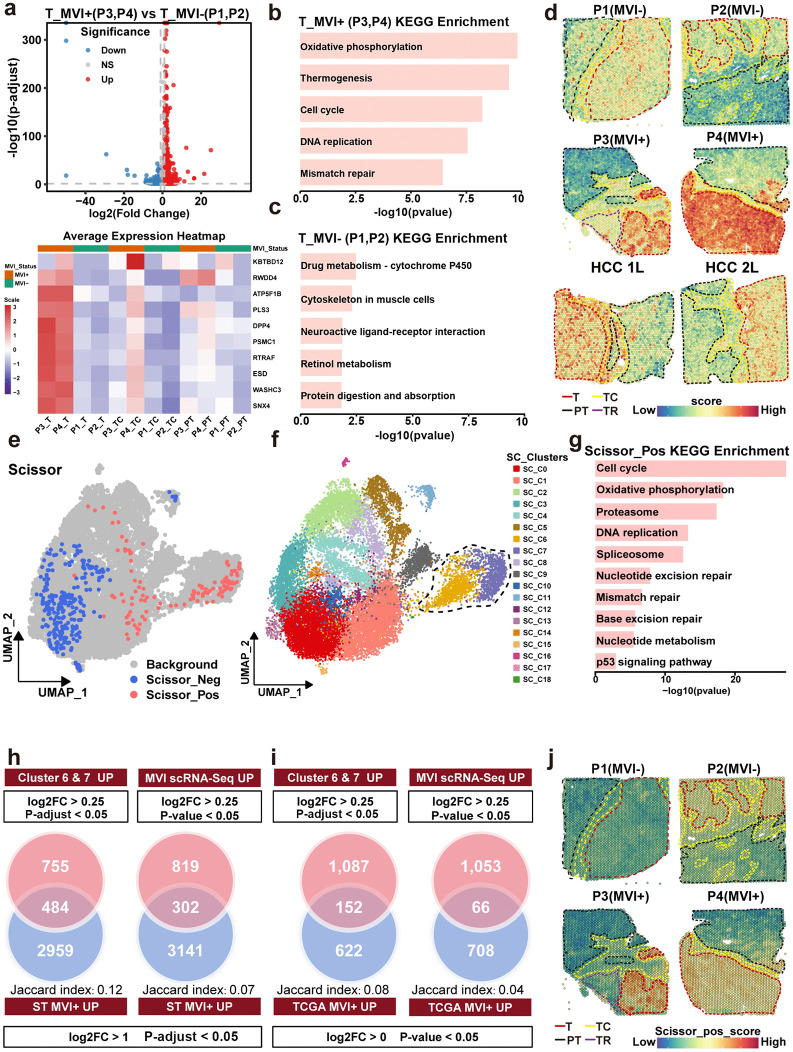
Regional transcriptional reprogramming in MVI+ tumors and identification of the corresponding single cell clusters. a, Volcano plot showing the differentially expressed genes (DEGs) in the T region between MVI+ and MVI−, and heatmap showing the top 10 DEGs. NS, no significance; MVI^+^, microvascular invasion positive; MVI^−^, microvascular invasion negative. **b and c**, Bar plot of Kyoto Encyclopedia of Genes and Genomes (KEGG) pathways enriched in DEGs of MVI^+^ T region (**b**) and MVI^−^ T region (c). **d**, Spatial feature plots showing the distribution of upregulated genes (UGs) scores. The UGs were upregulated genes in MVI^+^ compared with the MVI^−^ of TCGA-LIHC cohort. **e**, UMAP highlights the MVI^+^ phenotype in scRNA-Seq tumor cells of 79 HCC samples. Scissor_Positive (Scissor_Pos) represents MVI^+^ phenotype, while Scissor_Negative (Scissor_Neg) represents MVI^−^ phenotype. UMAP: Uniform manifold approximation and projection **f**, UMAP of clusters in scRNA-Seq tumor cells. The dotted circles indicate MVI^+^ specific clusters. **g**, Bar plot of KEGG pathways enriched in UGs of clusters SC_C6 and SC_C7. SC: Single cell. **h-i**, Comparison of UGs between the proliferation clusters SC_C6 and SC_C7 and MVI^+^ scRNA-Seq tumor cells of GSE242889. The UGs of these two datasets were intersected with those of spatial transcriptomics (ST) data (**h**) and TCGA-LIHC data (i). **j**, Spatial feature plots of UG scores of clusters SC_C6 and SC_C7 in MVI^+^ and MVI^−^ ST data. T, tumor region; PT, paratumor region; TC, tumor capsule region; TR, transition state region; scRNA-seq, single-cell RNA sequencing; TCGA, the cancer genome atlas; LIHC, liver hepatocellular carcinoma; HCC, hepatocellular carcinoma.

To identify cancer cell populations associated with the MVI^+^ phenotype, we utilized TCGA-LIHC dataset as the reference for phenotypic annotation. Samples were categorized as MVI^+^ or MVI^−^ based on clinical metadata. scRNA-Seq data from HRA001748, including 79 HCC samples [23], were analyzed using the Scissor algorithm [25], which integrated single-cell data with bulk transcriptomic profiles to identify phenotype-associated cell populations. Specifically, we first converted the MVI phenotype into a binary variable (MVI^+^ = 1, non-MVI = 0) and preprocessed the bulk gene expression matrix. For the scRNA-seq data, we randomly sampled 20% of cells from each sample to balance computational efficiency and representativeness. Then, we incorporated the MVI^+^/MVI^−^ phenotype into Scissor’s network-regularized sparse regression model (binomial family for binary phenotype), with the parameter alpha = 0.5 to identify cells correlated with MVI phenotype. Then, the results passed reliability significance test by 10-fold cross-validation and the permutation 100 times. Among the results, Scissor positive cells were defined as those significantly positively correlated with the MVI^+^ phenotype, while scissor negative cells were those negatively correlated with MVI^+^ phenotype. Finally, we counted the proportion of Scissor-positive cells in each scRNA-Seq clusters. Since the top two clusters SC_6/SC_7 with the highest proportion of positive cells were clustered together in UMAP (Fig 3f), they were defined as Scissor positive clusters. Similarly, clusters SC_0, SC_3, SC_4, and SC_15, which displayed the highest proportions of negative cells, were defined as Scissor negative clusters.

### Upregulated gene identification for MVI^+^ ST tumor region, MVI^+^ scRNA-Seq, Scissor positive clusters, and TCGA-LIHC MVI^+^ HCC

Upregulated genes (UGs) in the ST T region were identified as described in the “Differentially expressed gene analysis of ST” section (log2 fold change > 1, adjusted *P* value < 0.05). MVI⁺-associated UGs from scRNA-seq data were determined using the FindAllMarkers function in Seurat, comparing the malignant cell populations between MVI⁺ and MVI⁻ samples derived from GSE242889 (log2 fold change > 0.25, *P* value < 0.05). For TCGA-LIHC MVI ⁺ HCC samples, UGs were identified via the DESeq2 R package by contrasting MVI⁺ samples with MVI⁻ samples from the TCGA-LIHC cohort (log2 fold change > 0, *P* value < 0.05). UGs specific to Scissor-positive clusters (SC_C6/SC_C7) were detected using Seurat’s FindAllMarkers function, with SC_C6/SC_C7 clusters compared against Scissor-negative clusters from HRA001748 (log2 fold change > 0.25, adjusted *P* value < 0.05).

### Survival analysis

To assess the overall survival (OS) of gene and gene sets in the bulk liver cancer cohorts, we used the gene count or calculated the gene set score with GSVA package. Five cohorts were downloaded for the survival analysis, including TCGA-LIHC, ICGC-LIHC, GSE14520, GSE40873, and GSE54236 [26–28]. The univariate Cox regression analysis was performed to measure the prognostic effects of certain genes on TCGA-LIHC cohorts. The OS analysis for each cohort was performed by the R package survminer.

### Airflow-Assisted Desorption Electrospray Ionization-Mass Spectrometry Imaging (AFADESI-MSI)

The 10 µm slices adjacent to the ST sections were used for metabolites mass spectrometry imaging (MSI). These slices were thaw-mounted on positively charged desorption plates (Thermo Scientific, U.S.A.). Sections were stored at −80 °C prior to further analysis. Before MSI, they were desiccated first at −20 °C for 1 h and then at room temperature for 2 h. Moreover, an adjacent slice was preserved for H&E staining. MSI was implemented through the AFADESI-MSI platform (Beijing Weiduo Technology Co., Beijing, China) and the Q-Orbitrap mass spectrometer (Q Exactive, Thermo Scientific, U.S.A.) platform, following previously reported methods [29]. The experiment was carried out on both positive and negative ion modes, with their mass range from 70 to 1,000 *m*/*z* and a resolution of 20,000. The solvent composition was acetonitrile (ACN)/water (H_2_O) (8:2) in both positive and negative mode. The solvent flow rate was 1.5 µL/min. The transporting gas flow rate was 45 L/min, and the spray voltage was set at 7 kV. The distance between the sample surface and the sprayer, as well as from the sprayer to the ion transporting tube, was 3 mm. The automated gain control (AGC) target was set to 2E6, with a maximum injection time of 200 ms. The S-lens voltage was maintained at 55 V, and the capillary temperature was set to 350 °C. For MSI acquisition, a continuous scanning rate of 0.2 mm/s was applied along the x-axis of the sample section, with a vertical step of 100 µm in the y-axis direction.

### Metabolite preprocessing

Raw mass spectrometry (MS) data were initially transformed into the imzML format utilizing the imzMLConverter software [30]. Following this, a comprehensive data normalization process was undertaken, incorporating spectral smoothing, baseline reduction, peak picking, peak alignment, and peak filtering steps, all executed within the Cardinal software package [31]. The refined imzML data were then integrated into the pySM framework, which leveraged both the proprietary SmetDB (maintained by Shanghai Luming Biotechnology Co.) and the publicly accessible Human Metabolome Database (HMDB, accessible at https://www.hmdb.ca) for enhanced metabolite definition. Within this framework, the molecular weights of ions present in the imaging MS dataset were meticulously compared against those of metabolites recorded in the aforementioned databases, ensuring a mass accuracy threshold of less than five parts per million (ppm), a capability enabled by the Q-Exactive Orbitrap mass spectrometer. This rigorous comparison, coupled with the consideration of isotopic abundance patterns within the MS data, allowed pySM to assign a metabolite-signal match score to the queried adducts, operating at a false discovery rate of 0.1. Ultimately, this comprehensive preprocessing and analysis pipeline culminated in the generation of a normalized intensity matrix for ions, accompanied by their corresponding metabolite annotations, providing a robust foundation for downstream metabolic profiling and analysis.

### Clustering of spatial metabolites

We utilized Spatial Shrunken Centroids (SSC) clustering method to segment the MSI into different clusters [32]. It combines the spatial awareness from Spatially Aware (SA) and Spatially Aware Structurally Adaptive (SASA) distances with the statistical regularization and mixture modeling from Nearest Shrunken Centroids [33]. This integration enables the SSC method to accurately segment images while automatically selecting important spectral features and determining the optimal number of segments. SA distance uses Gaussian weights, where pixels farther from the center are assigned lower weights. This helps to account for local variations in intensity and spatial structure. SASA goes a step further by also considering the similarity between neighboring pixels and the center. Pixels that are less similar to the center are assigned lower weights, further enhancing the ability to capture local variations and avoid over-smoothing. We used a smooth radius *r* = 1, an initial number of clusters *k* = 15, and a sparse parameter *s* = 3 to perform SSC clustering on MSI data. This algorithm will optimize the final result with *k* as the maximum number of clusters.

### Multiplex immunofluorescence

To perform MIF, tissue slides were initially deparaffinized in xylene and sequentially rehydrated in graded ethanol. Endogenous peroxidase activity was quenched using 0.3% hydrogen peroxide, followed by antigen retrieval in citrate buffer and blocking with 5% bovine serum albumin (BSA). Primary antibodies—Anti-STMN1 (Boster, BM4300), Anti-HMGN2 (Boster, A04839-2), Anti-TUBB (Boster, M05613-4), and Anti-Glypican 3 (Abcam, ab95363)—were applied to the slides and incubated for 60 min in a humidified chamber at 37 °C. This step was followed by incubation with the appropriate horseradish peroxidase (HRP)-conjugated secondary antibodies. To remove residual antibodies between staining rounds, the slides were re-treated with citrate buffer. Finally, the slides were counterstained with DAPI solution for nuclear visualization, incubated at 37 °C for 10 min in the dark. Images were acquired and analyzed using the CaseViewer software.

### MIF co-localization and quantification

MIF images were analyzed using the HALO software platform (Indica Labs, USA). Threshold settings for *STMN1*, *HMGN2*, and *GPC3* channels were manually determined from representative positive controls. For each channel, the background fluorescence intensity was measured, and the cutoff was set at the background mean intensity plus three times its standard deviation, thereby minimizing background noise and ensuring detection of true positive signals. The same thresholds were applied uniformly across all slides. Background subtraction was performed using HALO’s rolling ball algorithm (radius = 15 pixels). Nuclei were segmented using DAPI fluorescence, with cytoplasmic expansion of 3 µm to approximate cell boundaries. Objects <25 µm^2^ or >200 µm^2^ were excluded to minimize artifacts. For quantification, positive cells were identified by thresholding, and co-localization was assessed at the single-cell level based on overlapping fluorescence signals.

The following metrics were calculated:

(1) **Triple-positive ratio (primary endpoint):**

$$\begin{array}{c} \text{Triple}-\text{positive}\;\text{ratio}\;\left(\%\right)\;=\;\frac{\text{N}\text{umber}\;\text{of}\;{STMN\text{1}}^{+}{HMGN\text{2}}^{+}{GPC\text{3}}^{+}\;\text{cells}}{\text{T}\text{otal}\;{GPC\text{3}}^{+}\text{cells}}\times \text{100}\% \end{array}$$

(2) **Double-positive ratios:**

$$\begin{array}{c} \text{STMN1}-\text{GPC3}\;\text{co}-\text{localization}\;\text{ratio}\;(\%)=\frac{\text{N}\text{umber}\;\text{of}\;{STMN\text{1}}^{+}{GPC\text{3}}^{+}\;\text{cells}}{\text{T}\text{otal}\;{GPC\text{3}}^{+}\text{cells}}\times \text{100}\% \end{array}$$

$$\begin{array}{c} \text{HMGN2}-\text{GPC3}\;\text{co}-\text{localization}\;\text{ratio}\;(\%)=\frac{\text{N}\text{umber}\;\text{of}\;{HMGN\text{2}}^{+}{GPC\text{3}}^{+}\;\text{cells}}{\text{T}\text{otal}\;{GPC\text{3}}^{+}\text{cells}}\times \text{100}\% \end{array}$$

### Supplementary methods

Detailed descriptions of additional analytical and experimental methods are provided in supplementary methods sections of S1 File. These include CNV inference, cell type deconvolution, cell interaction analysis, cell-type specific metabolites analysis, cell culture, fibroblast isolation, immunofluorescence, western blotting, RNA extraction and quantitative real-time polymerase chain reaction (qPCR) analysis, cell counting kit-8 (CCK8) assay, 5-Ethynyl-2′-deoxyuridine (EDU) assay, co-culture, migration and invasion assays, and enzyme-linked immunosorbent assay (ELISA).

## Results

### Spatial transcriptomic and metabolomic features of MVI^+^ and MVI^−^ HCC

HCC exhibits a distinct spatial structural organization. To explore the biological differences in tissue architecture between MVI^+^ and MVI^−^ HCC, we employed spatial transcriptomic and metabolomic data to annotate their tissue structures and examine the basic distribution patterns and characteristics of cells and metabolites within these regions (S1 Fig).

### Spatial transcriptomic map of MVI^+^ and MVI^−^ HCC

To characterize the ST landscape of MVI^+^ and MVI^−^ HCC tumors, we performed ST sequencing on four tumor tissues (two for MVI^+^, two for MVI^−^) from tumor boundary tissues and two distant peritumoral tissues (S1 Table). The analysis encompassed 21,920 spots with an average detection of 6,945 genes per sample. After uniform normalization and dimensionality reduction, 13 distinct transcriptional clusters were identified by the shared nearest neighbor clustering analysis (Fig 1a and 1b). These clusters demonstrated heterogeneous expression of some established hepatocyte markers (*HAMP, MT1X, MT1G*) [16], immune/stromal markers (*CD3D, IGHG1, COL1A1, ACTA2*), and HCC malignancy-associated markers (*LAMB3, SERPINA12, TUBB*) [17–19]. Annotation of the T regions in H&E images by pathology experts revealed distinct differences in the composition of clusters between MVI^+^ and MVI^−^ HCC. The T region of MVI^−^ contained ST_C1, ST_C6, and ST_C8, while that of MVI^+^ contained ST_C1, ST_C10, and ST_C12 (Fig 1c–1e). Spatial mapping demonstrated compartmentalized distribution patterns of canonical hepatocyte and tumor microenvironment (TME) markers (S2 Fig), reflecting high-degree cellular regionalization within HCC specimens.

As each spot in ST contained multiple cells, it is necessary to clarify the cellular composition of these transcriptional clusters. Cell-type deconvolution was performed using public HCC scRNA-seq data [16] via the Robust Cell-Type Decomposition (RCTD) algorithm [34] (S1 File). Quantitative estimation of cellular proportions within the 13 transcriptional clusters revealed distinct distributions of hepatocytes, malignant hepatocytes, CAFs, endothelial cells, B lymphocytes, T/ NK cells, and myeloid populations in different clusters (S3 Fig). For example, cluster ST_C3 was primarily composed of stromal and immune cells, whereas cluster ST_C10 contained only cancer cells. Inter-sample analysis demonstrated substantial heterogeneity in cellular composition, with the P2 tumor specimen exhibiting obvious myeloid infiltration compared with other specimens (Figs 1f and S3), indicating patient-specific immunological variation. To explore the cell type bias of different clusters, hierarchical clustering was performed using the cellular components in the clusters. Clusters ST_C3 and ST_C5 exhibited heterogeneous cellular admixtures dominated by CAFs, accompanied by diverse endothelial and immune components (Figs 1g and S3). These two clusters were located in the TC region (Fig 1c–1e), indicating that this region is a mixed region of stromal cells and immune cells. Besides TC clusters, the hierarchical clustering revealed other two distinct cellular ecosystems, namely normal hepatocyte-enriched clusters (Clusters ST_C2, ST_C4, ST_C9, ST_C11) and tumor cell-dominant clusters (Clusters ST_C1, ST_C6, ST_C8, ST_C10, ST_C12, ST_C13) (Fig 1g). Furthermore, a spatial colocalization analysis was conducted to investigate the associations among cellular components within the tissue. The spatial dependencies between CAFs, endothelial/immune cells, and tumor cells suggested that tumor cell growth may be influenced by the surrounding microenvironment (Fig 1h). By integrating these findings with histomorphological information from H&E staining, the clusters could be classified into three pathologically defined regions for subsequent analyses, including the T region (Clusters ST_C1, ST_C6, ST_C8, ST_C10, ST_C12, ST_C13), the TC region (Clusters ST_C3, ST_C5), and the PT region (Clusters ST_C2, ST_C4, ST_C9, ST_C11) (Fig 1i).

According to the above regional delineation, only cluster ST_C4 was present in both the T (in P1) and the PT (in P2, P3, P4) region. To examine the region annotation of these clusters, a CNV analysis was conducted across all clusters using the inferCNV [35] (S1 File). In Patient P1, clusters ST_C2 and ST_C9 demonstrated lower CNV scores than clusters ST_C1, ST_C4, ST_C6, and ST_C8 (S4a and S4e Fig). Notably, in Patients P2–P4, clusters ST_C2, ST_C4, and ST_C9 exhibited attenuated CNV scores relative to tumor-dominant clusters (Clusters ST_C1, ST_C6, ST_C8, ST_C10, ST_C12, ST_C13; S4b–S4e Fig). These differential CNV patterns revealed patient-specific malignant composition, where cluster ST_C4 belonged to the T region in patient P1, but was located in the PT region in other patients, while the division of other clusters was consistent between cellular deconvolution and CNV analysis. Therefore, we corrected the division of the T region and classified cluster ST_C4 in P1 into the T region. Cluster ST_C7, which was uniquely identified in Patient P3 (Fig 1d), displayed a gene expression pattern different from both the T and PT region. This cluster contained both normal hepatocytes and tumor cells (S4f Fig), suggesting its role as a transitional state between normal and malignant phenotypes. Given this ambiguous biological status, Cluster ST_C7 was excluded from subsequent comparative transcriptomic analyses between MVI^+^ and MVI^−^ HCC. Normal hepatocyte identity was confirmed for clusters ST_C1, ST_C2, ST_C4, ST_C6-13 in control specimens N1 and N3. Finally, spatial mapping identified distinct regions in all samples with TC serving as a structural interface between the T and PT regions (Fig 1i).

### Spatial metabolomics to elucidate the regional distribution of metabolites

Variations in transcriptional function are often accompanied by metabolic reprogramming. To systematically investigate the metabolic differences between MVI^+^ and MVI^−^ HCC, SM was conducted on six tissue specimens. This high-resolution approach detected 719 positive and 408 negative metabolites with molecular weights ranging from 74.09 to 1189.99 *m*/*z* and 73.03 to 1041.81 *m*/*z*, respectively (S3 Table). To investigate the spatial distribution patterns of metabolites, a clustering analysis was conducted on the metabolite profiles. SSC clustering revealed 15 distinct spatial clusters, where both positive and negative metabolites could successfully reconstruct the tissue architecture as visualized by H&E-stained images (Figs 2a, 2b, S5a, and S5b). This spatial distribution pattern demonstrated the region-specific aggregation of metabolites in HCC.

Hierarchical clustering of metabolites identified differential intensity patterns for positive metabolites across different clusters (Fig 2c; S4 Table; S1 File), whereas negative metabolites showed ambiguous inter-cluster variations (S5c Fig) and thus were excluded in further analyses. The above results indicated that the composition of metabolites varies substantially across different clusters. Spatial mapping revealed distinct metabolic regions: clusters SM_C2, SM_C10, SM_C13, and SM_C15 were preferentially localized in the T region of MVI^–^ but in the PT region of MVI^+^; while clusters SM_C9 and SM_C11 occupied the TC region across both phenotypes (Fig 2b–2d). Given this spatial segregation, subsequent analysis would be focused on the T-region metabolic disparities and TC-region conserved signatures. The metabolites in clusters SM_C2, SM_C10, SM_C13, and SM_C15 showed significant enrichment (Fisher’s exact test, *P* < 0.05) of ‘lipids and lipid-like molecules’ in the MVI^−^ T region, particularly glycerolipids such as triglycerides (TG) and diacylglycerols (DG) (Figs 2e, S5d, and S5e), whereas these lipids were markedly depleted in the MVI^+^ T region (Fig 2b–2d and S4 Table). Notably, the TC region from both MVI^+^ and MVI^−^ samples exhibited conserved accumulation of “organic acids and derivatives” accompanied by marked depletion of lipids (Fig 2f). Overall, these spatially resolved findings highlighted the metabolic heterogeneity within the TME of HCC, and also suggested that the major change in the T region may be related to glyceride metabolism.

Overall, both the spatial transcriptomic and metabolomic analyses revealed well-defined tissue regions. Therefore, the subsequent analyses would be focused on comparison of the T and TC regions in MVI^+^ and MVI^−^ HCC, respectively.

### MVI^+^ specific malignant cell population and its characteristics in MVI^+^ tumor foci

MVI is widely regarded as a pathological hallmark of aggressive tumor behavior and has been linked to pro-invasive processes in HCC [36,37]. Motivated by this established clinical-pathological observation, we attempted to use multi-omics datasets, including ST, scRNA-seq, TCGA bulk RNA-seq profiles and MIF, to dissect the core functional features and specific malignant subpopulation of MVI⁺ HCC tumor cells (S1 Fig). We also tried to explore the metabolic functional dynamics for this specific subpopulation by combining SM data (S1 Fig).

### The core malignant cell population of MVI^+^ was identified by combining bulk RNA-Seq, scRNA-Seq and ST data

To evaluate the transcriptomic variations, an analysis of DEGs was conducted on the T region of MVI^+^ and MVI^−^ tumors with the “devil” R-package (see Methods, the patient identity was used as a covariate). As a result, 3,443 upregulated DEGs and 326 downregulated DEGs were identified in MVI^+^ samples relative to the MVI^−^ samples (Fig 3a and S5 Table). The top 10 DEGs had higher expression in the MVI^+^ T region than in the MVI^−^ T region and other regions (Fig 3a), indicating reliability of the results of DEGs identification. KEGG pathway enrichment analysis identified that the pathways related to cell proliferation, such as “Cell cycle,” “DNA replication,” and “Mismatch repair,” were preferentially enriched in MVI^+^ tumors (Fig 3b and S6 Table). “Drug metabolism-cytochrome P450” and “Retinol metabolism,” were predominantly activated in MVI^−^ tumors (Fig 3c). These results indicated obvious molecular and functional differences between MVI^+^ and MVI^−^ tumor cells. Moreover, another DEGs analysis was conducted using the TCGA LIHC bulk RNA-Seq cohort to identify the upregulated DEGs set in MVI^+^ tumor. GSVA score analysis using this gene set revealed that MVI^+^ samples had higher GSVA scores (P3, P4, and the publicly available HCC-1L, HCC-2L) compared with the MVI^−^ samples (Fig 3d). These results demonstrated the robustness and accuracy of MVI^+^ and MVI^−^ ST datasets presented in this study and that MVI^+^ and MVI^−^ tumor cells have obvious functional differences.

To determine whether these functional differences in the T region arise from specific cell subtypes in MVI^+^ HCC, the Scissor algorithm was implemented to integrate TCGA with scRNA-seq profiles from a large single-cell cohort of 79 samples [23]. As a result, clusters SC_C6 and SC_C7 were identified as predominant MVI^+^ tumor-associated subpopulations (Fig 3e and 3f). Pathway enrichment analysis revealed that these clusters exhibited activation of proliferation-associated pathways, which is consistent with the findings from our ST analysis of MVI⁺ HCC samples (S6 Table; Fig 3b and 3g), further validating their functional correspondence to the MVI⁺ phenotype. To demonstrate the relevance of the SC_C6/SC_C7 clusters to MVI^+^, we compared the UGs in these clusters with those in MVI^+^ identified in another independent scRNA-Seq dataset (GSE242889) [10]. We calculated the Jaccard index for two sets of pairwise intersections: (1) SC_C6/SC_C7 UGs versus ST MVI⁺ UGs, and SC_C6/SC_C7 UGs versus TCGA-LIHC MVI⁺ UGs; (2) scRNA-seq MVI⁺ UGs versus ST MVI⁺ UGs, and scRNA-seq MVI⁺ UGs versus TCGA-LIHC MVI⁺ UGs. Notably, the Jaccard index generated from SC_C6/SC_C7 UGs intersections was consistently higher than that generated from scRNA-seq MVI⁺ UGs intersections (Fig 3h and 3i). This suggests that the SC_C6/SC_C7 UGs are more representative of the MVI-positive phenotype than the scRNA-seq MVI⁺ UGs of GSE242889. Furthermore, the spatial map for the scores of SC_C6/SC_C7 UGs further confirmed that these subpopulations were preferentially localized within the MVI^+^ T region relative to the MVI^−^ T region, and the T region had higher signature scores than the TC and PT regions (Fig 3j). Collectively, these results established clusters SC_C6 and SC_C7 as reliable MVI^+^ specific subpopulations that encode the hallmark biological features of MVI^+^ HCC.

**Fig 4:**
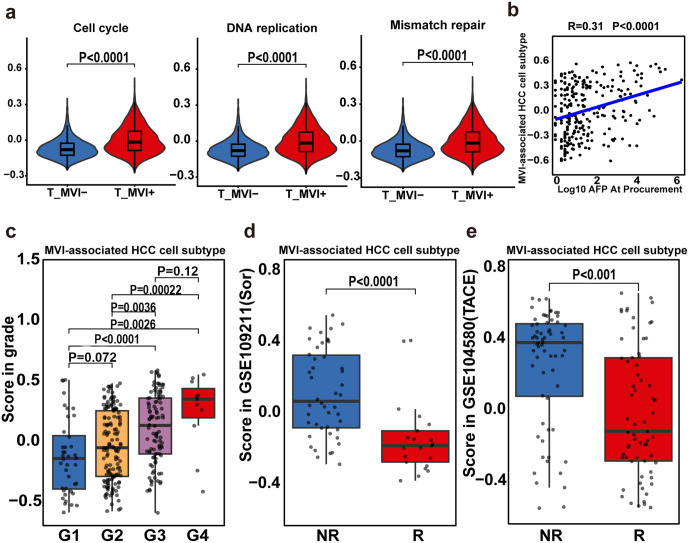
Transcription difference of T region between MVI+ and MVI− HCC tumors and clinical relevance of the proliferation cell population. a, Violin plots of gene expression scores of three pathways between MVI+ and MVI− scRNA-Seq tumor cells, scRNA-seq: single-cell RNA sequencing, T: tumor region. b, Spearman correlation between gene expression score of MVI-associated HCC cell subtype and serum AFP level, AFP, Alpha-Fetoprotein. **c**. Gene expression score comparison of MVI-associated HCC cell subtype DEGs between tumor grades in TCGA-LIHC, TCGA, the cancer genome atlas; LIHC, liver hepatocellular carcinoma; DEGs, differentially expressed genes. **d**, Boxplot showing the gene set score of MVI-associated HCC cell subtype in Sorafenib (Sor) therapeutic response and non-response groups. **e**, Boxplot showing the gene set score of MVI-associated HCC cell subtype in transcatheter arterial chemoembolization (TACE) therapeutic response (R) and non-response groups (NR). MVI^−^, microvascular invasion negative; MVI^+^, microvascular invasion positive; HCC, hepatocellular carcinoma. Statistical analysis was performed using the Student *t* test (a, c, d, and e).

Given that MVI^+^ T regions exhibited activated tumor-associated functions (Fig 4a), we next determined whether clusters SC_C6 and SC_C7 have clinical relevance. A significant positive correlation (*r* = 0.31, *P* < 0.0001) was observed between these MVI^+^ tumor-associated subpopulations and the serum alpha-fetoprotein (AFP) level (Fig 4b), and these cellular clusters exhibited progressive growth with advancing tumor grades (Fig 4c), implying their potential roles in driving the oncological progression of HCC. To draw more therapeutic implications, a treatment response analysis was conducted across cohorts receiving systemic chemotherapy or transarterial chemoembolization (TACE). Notably, treatment-refractory patients displayed elevated MVI^+^ tumor-associated subpopulations compared with patients with a treatment response (Fig 4d and 4e), suggesting the utility of these two clusters as prognostic biomarkers for therapeutic resistance.

### *STMN1*^*+*^*HMGN2*^*+*^*GPC3*^*+*^ triple-positive marker defines the core malignant cell population of MVI^+^ HCC

To gain deeper insights into the functional features of the MVI^+^ associated subpopulation, we characterized the specific marker genes and explored its interactions with the TME.

**Fig 5:**
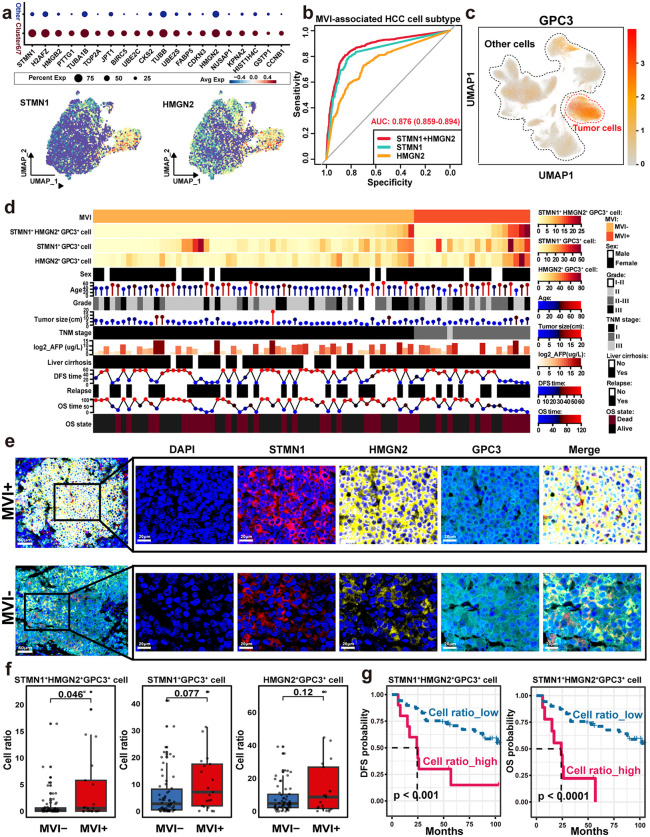
Clinical relevance of *STMN1+HMGN2+GPC3+* cell subtype in MVI^+^ HCC. a, Dot plot of marker genes in MVI-associated HCC cell subtype and UMAP of marker genes (STMN1 and HMGN2) expression in clusters SC_C6 and SC_C7. Other represents clusters SC_0, SC_3, SC_4 and SC_15, UMAP: Uniform Manifold Approximation and Projection, MVI, microvascular invasion. **b**, Receiver operating characteristic curves of gene combinations predicted by the random forest model. AUC, area under the curve; HCC, hepatocellular carcinoma. **c**, UMAP showing the expression of marker gene *GPC3* in tumor cells and other cells in 79 MVI^+^ and MVI^−^ samples. **d**, Heatmap showing the distribution of clinical indicators and the proportion of *STMN1*^*+*^*HMGN2*^*+*^*GPC3*^*+*^ cell subtype in 79 MVI^+^ and MVI^−^ samples. DFS, disease-free survival; OS, overall survival; AFP, alpha-fetoprotein; TNM, tumor node metastasis. **e**, Multiplex immunofluorescence of *STMN1*, *HMGN2* and *GPC3* in MVI^+^ and MVI^−^ samples. MVI^+^, Microvascular invasion positive; MVI^−^, microvascular invasion negative. **f**, Proportions of *STMN1*^*+*^*HMGN2*^*+*^*GPC3*^*+*^ cell subtype, *STMN1*^*+*^*GPC3*^*+*^ cell subtype and *HMGN2*^*+*^*GPC3*^*+*^ cell subtype in 79 MVI^+^ and MVI^−^ samples. **g**, DFS and OS of *STMN1*^*+*^*HMGN2*^*+*^*GPC3*^*+*^ cell subtype proportion. DFS, disease-free survival; OS, overall survival. Statistical analysis was performed using the Student *t* test (f) or log-rank test (g).

DEGs analysis identified signature biomarkers such as *STMN1, HMGN2*, and *TUBB* for clusters SC_C6 and SC_C7 (Fig 5a and S7 Table). Cell-specific analysis of these marker genes identified *STMN1* as the marker gene with the highest subgroup specificity. Moreover, *HMGN2* displayed a strong correlation with *STMN1* in the TCGA-LIHC RNA-Seq dataset (S6a Fig, see Methods). Our results demonstrated that high expression levels of *STMN1* and *HMGN2* are closely related to advanced tumor stage and unfavorable clinical outcomes in HCC (S6b and S6c Fig). Furthermore, a combinatorial analysis demonstrated that dual *STMN1/HMGN2* expression provided a superior discriminatory power (AUC = 0.87) in detecting MVI-associated subpopulations (Fig 5b). Therefore, we used these two genes as representative genes of clusters SC_C6/ SC_C7. To enhance tumor-specific identification of MVI-associated subpopulations, the established HCC biomarker *GPC3*, which was mainly distributed in malignant cells (Fig 5c), was integrated with *STMN1* and *HMGN2*, resulting in the definition of a triple-positive (*STMN1⁺HMGN2⁺GPC3⁺*) malignant phenotype with high clinical specificity. Moreover, MIF validation across 79 HCC specimens (21 MVI⁺, 58 MVI⁻) revealed enrichment of *STMN1⁺HMGN2⁺GPC3⁺* cells in MVI⁺ tumors (Figs 5d, 5e, S6d, and S6e). A further quantitative analysis indicated that while there was no statistical significance in individual *STMN1* (*P* = 0.077) or *HMGN2* (*P* = 0.12) expression, the enrichment of *STMN1⁺HMGN2⁺GPC3⁺* cells exhibited a robust association with the MVI status (*P* = 0.046) (Fig 5f). Survival correlation analysis further identified elevated *STMN1⁺HMGN2⁺GPC3⁺* abundance as an independent prognostic factor, which was significantly associated with reduced OS (hazard ratio (HR) = 1.2, 95% confidence intervals (CI) [1.1, 1.2], *P < 0.0001*) and DFS (HR = 1.1, 95% CI [1.1, 1.2], *P < 0.001*) (Fig 5g). These results suggested that *STMN1⁺HMGN2⁺GPC3⁺* can be used to define MVI^+^ associated malignant cell subpopulations.

We further explored the functional characteristics of this cell subpopulation from the perspective of cell interactions. Intercellular communication mapping revealed differential pathway activation between the *STMN1*^*+*^*HMGN2*^*+*^*GPC3*^*+*^ cell population and other tumor cell populations in the 79 scRNA-Seq samples, including previously reported *MDK-NCL* axis [10] and the newly found *PPIA-BSG* signaling (S7a–S7c Fig). ST and immunofluorescence colocalization confirmed preferential *PPIA-BSG* signaling in MVI⁺ tumors (S7d and S7e Fig). Clinical-stage correlation analysis demonstrated progressive *PPIA/BSG* upregulation in advanced-stage tumors, with both markers showing significant (*P < 0.05*) survival associations (S7f and S7g Fig), suggesting their functional roles in MVI-related HCC progression. Collectively, these results identified *STMN1⁺ HMGN2⁺ GPC3⁺* cell subtype as an MVI^+^ specific cell subpopulation, which is specifically correlated with MVI progression and characterized by distinct interaction networks and clinical relevance, thereby improving our mechanistic understanding of MVI- related HCC progression.

### Glycerolipid reprogramming is a hallmark of MVI^+^ tumor lesions and is downregulated in *STMN1*^*+*^*HMGN2*^*+*^*GPC3*^*+*^ cell subtype

We then integrated spatial transcriptomic and spatial metabolomic data to further investigate the molecular features of the *STMN1*^*+*^*HMGN2*^*+*^ *GPC3*^*+*^ cell subtype.

**Fig 6:**
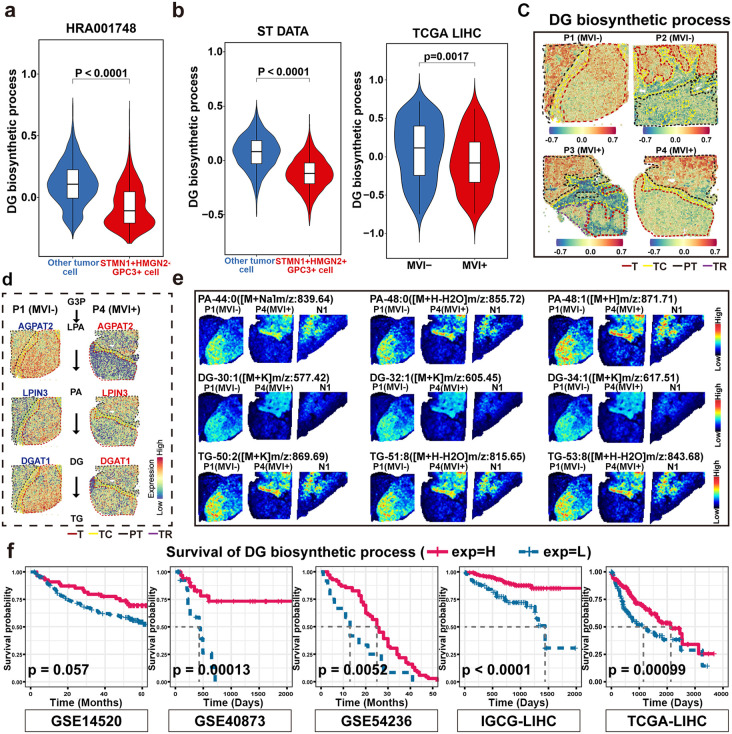
Diacylglycerol biosynthesis decreased in MVI^+^ tumor region and *STMN1+HMGN2+GPC3+* cell subtype. **a**, Comparison of the “diacylglycerol (DG) biosynthesis process” pathway score between *STMN1*^*+*^*HMGN2*^*+*^*GPC3*^*+*^ cell subtype and other HCC tumor cells. **b**, Violin plots showing the gene expression score of “diacylglycerol biosynthesis process” pathways in the T region of MVI^+^ and MVI^−^ HCC (left) and TCGA-LIHC MVI^+^ and MVI^−^ HCC (right). ST, spatial transcriptomics; TCGA, the cancer genome atlas; LIHC, liver hepatocellular carcinoma; MVI^+^, microvascular invasion positive; MVI^−^, microvascular invasion negative. **c**, Spatial plot of “diacylglycerol biosynthesis process” pathway scores in MVI^+^ and MVI^−^ tissue regions. T, tumor region; PT, paratumor region; TC:, tumor capsule region and TR, transition state region. **d**, Enzymes and metabolites involved in TG biosynthesis. G3P, glyceraldehyde-3-phosphate; LPA, lysophosphatidic acid; PA, phosphatidic acid; and TG, triglyceride. **e**, Spatial images of PA, DG, and TG in P1, P4, and N1 tissues. **f**, Overall survival based on “diacylglycerol biosynthetic process” pathway score in five liver cancer cohorts. H, high; L, low. Statistical analysis was performed using the Student *t* test (a and b) or log-rank test **(f)**.

Since there were obvious differences in glyceride-related metabolites in the T region between MVI^+^ and MVI^−^ (Fig 2b and 2e), we first analyzed the activity of glyceride-related pathways in the gene transcriptome. Notably, the *STMN1*^*+*^*HMGN2*^*+*^ *GPC3*^*+*^ cell subtype exhibited significant (*t* test, *P* < 0.0001) downregulation of relevant metabolic pathways, including “diacylglycerol biosynthesis” and “triglyceride biosynthetic processes” (Figs 6a and S8c). Pan-dataset validation using TCGA-LIHC and ST consistently revealed that the expression of gene sets associated with these pathways was suppressed in MVI^+^ cohort relative to that in MVI^−^ cohort (Figs 6b, 6c, S8a, and S8b), further supporting their potential roles in tumor progression. These results indicated that reduced activity of glyceride metabolism-related pathways at the transcriptional level is a distinctive feature of the triple-positive cell population.

Furthermore, we conducted an integrated analysis on the key enzymes and corresponding metabolites within the triglyceride biosynthetic process. The MVI^−^ T region showed elevated expression levels of enzymes critical for the conversion of lysophosphatidic acid (LPA) to TG compared with the MVI^+^ T region, including *AGPAT2*, *LPIN3*, and *DGAT1* (Fig 6d). Spatial metabolite mapping further corroborated the above result, demonstrating regional accumulation of pathway intermediates including phosphatidic acid (PA), DG, and TG in the MVI^−^ T region (Fig 6e). To facilitate regional quantitative comparisons of metabolism, we established a computational framework by aligning ST-derived spatial annotations with the histopathological landmarks (S8b Fig). The results further revealed that the MVI^+^ T region displayed markedly lower PA, DG, and TG signal intensities than the MVI^−^ T region. Collectively, these results suggested progressive attenuation of glycerolipid biosynthesis during the transition from MVI^−^ to MVI^+^.

Furthermore, clinical relevance analysis further confirmed the importance of diacylglycerol biosynthesis in MVI as their reduced activity corresponded to poorer OS across five independent cohorts (Fig 6f). Therefore, TG biosynthesis impairment, particularly at the diacylglycerol node, may be a hallmark metabolic alteration in MVI-related HCC progression.

### Key cellular and metabolic dysfunction in MVI^+^ tumor capsule region

Besides the malignant cells within the tumor lesion, the microenvironment of the TC also plays a critical role in HCC growth by acting as a physical barrier and serving as a key site for cellular and metabolic interactions to influence tumor progression. However, the differences between MVI^+^ and MVI^−^ tumors in this region remain unclear. To investigate the functional roles of cells and metabolites within the TC, we compared the transcriptional and metabolic differences, performed spatial correlation analyses between capsule metabolites and cellular components using ST, and further validated the influence of key cells and metabolites on tumor growth through in vitro experiments.

### Inhibition of iCAFs in the capsule region may be associated with the progression of MVI^+^ HCC

**Fig 7:**
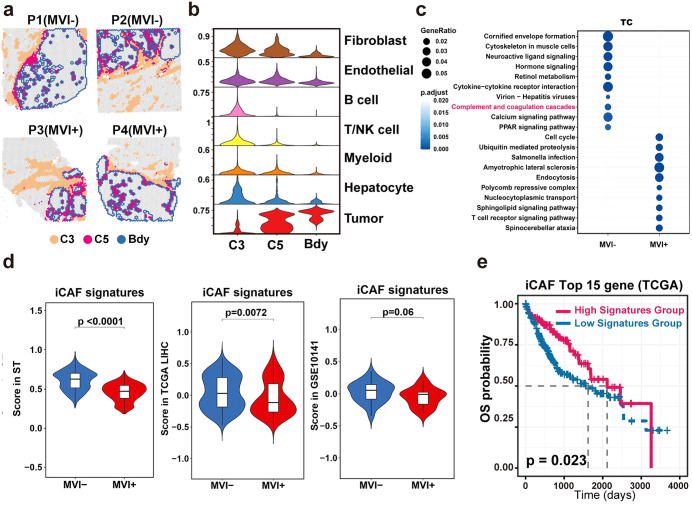
iCAF suppression in the MVI^+^ HCC capsule. **a**, Spatial feature plots of cluster ST_C3 (C3), cluster ST_C5 (C5), and tumor boundary (Bdy) for all patients. ST, spatial transcriptomics. **b**, Violin plots of cell proportions in C3, C5 and Bdy. NK, Natural killer. **c**, Dot plot of KEGG pathway enrichment for DEGs of the TC region between MVI^+^ and MVI^−^ tumors. MVI^+^, microvascular invasion positive, MVI^−^, microvascular invasion negative, TC, tumor capsule region. **d**, iCAF signature score comparison between MVI^+^ and MVI^−^ in ST, TCGA-LIHC, and GSE10141 data. iCAF, inflammatory cancer-associated fibroblast; TCGA, the cancer genome atlas, LIHC, liver hepatocellular carcinoma. **e**, Overall survival (OS) plot of top 15 iCAF marker genes in TCGA-LIHC patients. Statistical analysis was performed using the Student *t* test (d) or log-rank test (e).

To elucidate the functional characteristics of the TC region, we first compared the gene expression in the TC region between MVI+ and MVI^−^. The TC region consisted of cluster ST_C5 and cluster ST_C3 (Fig 7a), which were identified as corresponding to the tumor capsule (Fig 1c, 1d, and 1i). Both clusters had high expression of the marker genes Collagen Type I Alpha 1 Chain (*COL1A1*) and Collagen Type I Alpha 2 Chain (*COL1A2*) of CAFs (Fig 1b). Cluster ST_C5, which was located at the leading edge of the TC region and adjacent to the tumor foci (Fig 7a), demonstrated higher expression of Actin Alpha 2, Smooth Muscle (*ACTA2*) and Myosin Heavy Chain 11 (*MYH11*) (Fig 1b), two markers indicative of vascular cancer-associated fibroblasts [24]. Specifically, compared with the tumor boundary, clusters ST_C5 and ST_C3 showed a sequential increase in the number of fibroblasts (Fig 7b), suggesting possible spatial heterogeneity in the distribution of CAF subpopulations. Subsequent DEGs profiling combined with KEGG pathway analysis between MVI^+^ and MVI^−^ specimens revealed notable pathway dysregulation in the TC region. The “Complement and coagulation cascades” pathway was significantly (*P.adjust < 0.05*) suppressed in the MVI^+^ TC region relative to that in the MVI^−^ TC region (Fig 7c), which has been previously documented in iCAF populations within the TME [38]. This suppression implied functional impairment of iCAFs in MVI^+^ HCC.

To validate the above possibility, we performed spatial transcriptomic quantification of iCAF signature scores [24] for spots in the TC region. The results revealed that the MVI^+^ TC region had substantially lower iCAF signature scores than the MVI^−^ TC region (Fig 7d), which was further corroborated by two independent validation cohorts (TCGA-LIHC and GSE10141). TCGA-LIHC cohort analysis further identified a pronounced association between elevated iCAF signature scores and enhanced clinical outcomes (Fig 7e). These results collectively indicated that the downregulation of iCAF-associated genes may facilitate MVI-related HCC progression.

Next, we explored whether the functional changes of iCAFs in MVI^+^ HCC are associated with specific metabolites. First, we found that iCAFs exhibit a distinct spatial distribution pattern, mainly localized in regions distal to the tumor foci (S9 Fig and S1 File). Second, this distribution of iCAFs shows a spatial correlation with taurine, a metabolite highly expressed in the TC region of MVI^+^ HCC (S10–S13 Figs; S1 File; S8 and S9 Tables). Finally, in vitro induction and co-culture assays reveal that while taurine exhibits no direct effect on the proliferation of HCC cells, it significantly suppresses the proliferation and characteristic marker expression of iCAFs, thereby abrogating their inhibitory effect on HCC cell migration and invasion (S10 and S14–S17 Figs; S1 File; S10 Table). Collectively, this suggests that taurine drives malignant HCC progression by indirectly reprogramming iCAF functions rather than directly targeting tumor cells. Detailed descriptions of this part of the content are provided in the S1 File.

## Discussion

MVI is closely associated with the recurrence and metastasis of HCC [39], underscoring the urgent need to elucidate the molecular mechanisms driving the progression of MVI^+^ HCC. In the present study, we systematically characterized the spatial transcriptional and metabolic landscapes of both tumor lesion and capsular regions during MVI development. The *STMN1⁺HMGN2⁺GPC3⁺* cell subtype was specifically enriched in the T region of MVI^+^ HCC, exhibited distinct downregulation of glycerolipid metabolism, and was strongly associated with MVI-related HCC progression. In the TC region, taurine exhibited a strikingly consistent spatial distribution with iCAFs, and promoted MVI in HCC by suppressing the anti-tumor function of iCAFs.

MVI^+^ malignant cell populations were reported to be characterized by highly proliferative features in previous single-cell and multi-omics studies [10,40]. Consistently, the *STMN1⁺HMGN2⁺GPC3⁺* cell subtype identified in this study also exhibited pronounced proliferative capacity, and the number of infiltrating triple-positive malignant cells was associated with poor prognosis in patients with HCC. Notably, different from previously reported results for MVI^+^ malignant populations, *HMGN2* and *STMN1* were specifically recognized as molecular markers of this MVI-associated cell subpopulation. *STMN1* encodes a phosphoprotein that disrupts microtubule dynamics by sequestering α/β-tubulin dimers, thereby promoting microtubule destabilization and driving tumor cell proliferation, migration, and differentiation [25]. *STMN1*, which is markedly upregulated in MVI^+^ HCC, can be used to predict the differentiation trajectory of HCC cells, thereby contributing to pathological diagnosis and prognosis in clinical practices [41,42]. *HMGN2*, a member of the high-mobility group family, binds nucleosomes and regulates gene transcription by modulating histone post-translational modification. A recent study has indicted that *HMGN2* accelerates tumor cell proliferation and cell cycle progression by regulating *CDC20* expression [26]*. HMGN2* has been linked to poor overall and relapse-free survival in patients with HCC [43]. *GPC3*, which is overexpressed in 70%–80% of HCCs and absent in normal liver, is a cell-surface heparan sulfate proteoglycan and participates in a variety of processes such as cell growth, differentiation, and migration [44,45]. Its specific distribution in tumors and crucial role in tumor development make it a promising target in the diagnosis and treatment of HCC [46]. Notably, several *GPC3*-based immunotherapies have already been adopted in clinical trial pipelines [47]. These facts further reinforce the clinical significance of the *STMN1⁺HMGN2⁺GPC3⁺* cell subtype in HCC. Furthermore, we also observed that metabolic pathway enrichment was present in the tumor regions of MVI^-^ HCC. It has been reported that the “Drug metabolism-cytochrome P450” and “Retinol metabolism” pathways and the involved cytochrome P450s enzymes are downregulated during HCC progression [48,49].

In agreement with the impaired metabolic activity of malignant cells associated with vascular invasion of HCC [50], the *STMN1⁺HMGN2⁺GPC3⁺* cell subtype exhibited a metabolically repressive phenotype, particularly evidenced by the downregulation of glycerolipid metabolism. Specifically, TG and DG, two important components of glycerolipid, decreased in the MVI^+^ T region compared with those in the MVI^−^ T region. Moreover, the DG biosynthesis pathway is positively associated with improved prognosis of HCC across multiple external cohorts, suggesting its protective role in glycerolipid-related pathways during MVI transition. This protective role may stem from glycerolipid hydrolysis products, such as polyunsaturated fatty acids, which induce ferroptosis in tumor cells through lipid peroxidation [51]. Additionally, lipid metabolism is intricately regulated by cytokines [52]. During MVI transition, MVI^+^ tumor cells will secrete more proinflammatory cytokines, such as *TNF-α* and *IL-6*, which suppress glycerolipid synthesis [53–55] and promote tumor cell invasion [56,57]. Thus, further research is needed to elucidate the molecular mechanisms underlying the suppression of glycerolipid metabolism in MVI-related HCC.

The *STMN1⁺HMGN2⁺GPC3⁺* cell subtype was identified to communicate with fibroblasts via the binding of *PPIA* to receptor *BSG*. Under pro-inflammatory stimulation, *PPIA* can be secreted by cells through the autocrine or paracrine pathways [58,59]. *PPIA* is commonly upregulated in multiple tumors and involved in pathological processes including proliferation [60], cell cycle [61], apoptosis resistance [62], and invasion capacity [63]. For example, *PPIA* can enhance the production of matrix metalloproteinase (*MMP*) and pro-inflammatory cytokines, thereby promoting Th1-type immune responses, remodeling the TME, and facilitating metastasis [64]. *BSG* is a main receptor of *PPIA* and activates intracellular oncogenic related signaling pathways in receptor cells upon ligand binding [65,66]. Specifically, tumor-derived *PPIA* binds to *BSG* on neutrophils, triggering neutrophil extracellular trap (NET) formation and elastase release to drive breast cancer progression and invasion [67]. Notably, *STMN1* activates the transcription factor STAT3 [68], which enhances *PPIA* transcription [69]. Moreover, *HMGN2* is positively involved in *NF-κB* activation [70], which is another pathway mediating *PPIA* upregulation [71]. These findings suggest that the *STMN1⁺HMGN2⁺GPC3⁺* cell subtype may represent an important source of *PPIA*, and targeting this specific cell population or blocking *PPIA* secretion may offer a potential therapeutic strategy for MVI-related HCC.

The TC region is characterized by highly heterogeneous cell types, including CAFs, endothelial cells, T cells, B cells, and myeloid cells. Interestingly, the spatial distribution of different CAF subtypes is particularly distinctive. The mCAF density decreases whereas the iCAF and apCAF densities increase with distance from the tumor, which is similar to their distribution in pancreatic cancer [72]. Notably, iCAFs have been reported to have antitumor functions and be associated with favorable prognosis in several cancers, including pancreatic cancer and HCC [73,74]. Cheng and colleagues classified the CAFs in HCC into mCAFs and *C7*-CAFs, with the latter closely resembling the iCAFs identified in this study. They further demonstrated that the *C7*-CAF subpopulation is potentially regulated by USF2, as knockdown of USF2 reduced the expression of *C7*-CAF signature genes, which was accompanied by enhanced inflammatory responses, chemotaxis, and cell migration. Therefore, they concluded that diminished transcriptional activity within the *C7*-CAF subpopulation contributes to tumor progression [73]. In the present study, we observed suppressed iCAF activity in the MVI^+^ TC region, which was further validated in two independent bulk RNA-seq and microarray cohorts [75,76], underscoring the clinical significance of iCAFs in HCC. It should be particularly noted that taurine was spatially correlated with iCAFs and promoted HCC metastasis by suppressing iCAF activity.

Taurine is a sulfur-containing amino acid that predominantly exists in a free form in mammals and plays critical roles in various physiological and pathological processes, including aging, obesity, and cancers [77–79]. Although taurine has been reported to enhance the efficacy of immunotherapy by augmenting CD8⁺ T cell cytotoxicity, tumor cells can promote taurine efflux from tumor-associated macrophages and competitively absorb extracellular taurine via the taurine transporter *SLC6A6* to support their proliferation and metastasis [80–82]. Nevertheless, taurine showed no direct effect on tumor cells in this study. This phenomenon may be due to the relatively low *SLC6A6* expression in HCC cells, which may limit taurine uptake and attenuate its direct effect. Such transporter-dependent differences may contribute to the distinct role of taurine observed in HCC.

A major limitation of this study is the small sample size of spatial multi-omics data, which included only two patients with MVI⁺ and two patients with MVI⁻. Although we have employed statistical methods optimized for small sample sizes to mitigate the potential bias, the limited sample size may still affect the robustness of the conclusions. To address this inherent limitation and enhance the credibility of our findings, we have integrated multiple omics validation strategies, including publicly available scRNA-seq datasets, bulk RNA sequencing data, MIF assays, and in vitro cell function experiments. Notably, metabolic reprogramming is a hallmark of malignant cell subtypes in HCC [83,84]. The reduced metabolic activity of malignant cells in patients with MVI⁺ HCC may not be limited to glycerolipid metabolism. A direct comparison of metabolic profiles from malignant cells isolated from a larger cohort of MVI⁺ and MVI⁻ patients can not only validate our conclusions but also reveal broader metabolic dysregulations associated with MVI⁺ HCC. Similarly, the functional variations in iCAFs require further confirmation and investigation with a larger sample size. Therefore, future studies utilizing single-cell or spatially resolved multi-omics technologies on larger cohorts are crucial for validating, refining, and expanding the mechanistic insights proposed in this study.

In summary, this study integrated ST, SM, and single-cell data to provide a comprehensive spatial atlas of MVI^+^ HCC. First, within the T region, the *STMN1*^*+*^*HMGN2*^*+*^*GPC3*^*+*^ cell subtype was identified and characterized by a highly proliferative phenotype, which may be responsible for the poor prognosis in patients with HCC. Second, the *STMN1*^*+*^*HMGN2*^*+*^*GPC3*^*+*^ cell subtype was specifically accumulated in the MVI^+^ T region and displayed a suppression of glycerolipid metabolism. Additionally, glycerolipid biosynthesis pathways are correlated with improved prognosis in HCC, highlighting the protective role of glycerolipid metabolism. In the TC region, iCAFs were primarily distributed distally from the tumor foci and notably suppressed in MVI-related HCC. This suppression was strongly correlated with the taurine level, and subsequent validation revealed that taurine could promote HCC progression by inhibiting iCAFs. The present study provides new insights into the transcriptional and metabolic features of MVI-related HCC, offering valuable guidance for the development of more effective therapeutic strategies targeting MVI^+^ HCC.

## Supporting information

S1 Raw Images Original blot and gel images corresponding to the supplementary figures. (PDF)

S1 File Supplementary results and methods. (DOCX)

S1 Fig Graphic overview of this study design. ST, spatial transcriptomics; DEG, differentially expressed gene; MIF, multiplex immunofluorescence; WB, western blot; qPCR, quantitative real-time polymerase chain reaction; CCK8, cell counting kit-8; EDU, 5-ethynyl −2′- deoxyuridine; TCGA, the cancer genome atlas; LIHC, liver hepatocellular carcinoma; MVI^+^, microvascular invasion positive; MVI^−^, microvascular invasion negative; TC, tumor capsule region; CAF, cancer-associated fibroblast; iCAF, inflammatory cancer-associated fibroblast; qPCR, quantitative polymerase chain reaction; ELISA, enzyme-linked immunosorbent assay; HCC, hepatocellular carcinoma; T, tumor; scRNA-seq, single-cell RNA sequencing.
(TIF)

S2 Fig Marker genes expression features of each sample. The spatial feature plots showing the expression distributions of *NUPR1*(Tumor), *FABP1*(Hepatocytes), *COL1A1* (Fibroblasts), *CD68* (myeloids), *CD79A* (B cells), *IGHG1* (Plasma cells), *VWF* (endothelials), and *CD3E* (T cells) in each sample.
(TIF)

S3 Fig Cell proportions for clusters in each sample. The violin plots showing cells (hepatocytes, malignant hepatocytes (tumor), Fibroblasts, endothelial cells, B cells, T/NK cells and myeloid cells) proportion in the integrated clusters for each sample. NK, Natural killer; ST, spatial transcriptomics.
(TIF)

S4 Fig Copy number variation (CNV) of tumor samples and cell distribution in P3. **a–d**, Heatmap showing the CNV on chromosomes of tumor samples. **e**, Violin plot of CNV score in ST clusters in 4 tumor samples. **f**, Spatial distribution map of the seven predominant cell types in sample P3, alongside the H&E stained image. The red triangle denotes the TR region (Cluster ST_C7), indicative of a transitional state from normal to tumor. H&E, hematoxylin and eosin; ST, spatial transcriptomics; NK, Natural killer; TR, transition state region; MVI^+^, Microvascular invasion positive; MVI^−^, Microvascular invasion negative.
(TIF)

S5 Fig Spatial metabolism segmentation in all samples. **a**, Overview of spatial metabolism clusters for positive ions in N1 and N3 samples. **b**, Overview of spatial metabolism clusters for negative ions in each sample. **c**, Heatmap plot showing top 10 negative ions intensity in each cluster. **d and e**, The odds ratio of metabolites counts in tumor region (clusters SM_C2, SM_C10, SM_C13 and SM_C15) over that in the whole tissue by class (**d**) and sub class (**e**). Red dots represent a *P-value* of Fisher’s test <0.05, while blue dots represent a *P-value* of Fisher’s test >0.05. SM, spatial metabolomics; OR, odds ratio; CI, confidence interval; MVI^+^, microvascular invasion positive; MVI^−^, microvascular invasion negative.
(TIF)

S6 Fig Multiplex immunofluorescence of marker genes. a, Correlation between STMN1 and HMGN2 genes. TPM, transcripts per million. **b**, Overall survival of *STMN1* and *HMGN2* genes score in HCC cohort. **c**, Violin plot of *STMN1* and *HMGN2* genes expression scores of tumor stages, **d and e**, Multiplex immunofluorescence of *STMN1*^*+*^*GPC3*^*+*^ (**d**) and *HMGN2*^*+*^*GPC3*^*+*^ (**e**) cells in MVI^+^ and MVI^−^ samples. HR, hazard ratio; MVI^−^, microvascular invasion negative; MVI^+^, microvascular invasion positive.
(TIF)

S7 Fig Ligand-receptor difference between *STMN1*+*HMGN2*+*GPC3*+ cell subtype and TME cells. **a**, Bubble Heatmap showing the mean interaction strength between *STMN1*^*+*^*HMGN2*^*+*^*GPC3*^*+*^ cell subtype and other tumor cells for ligand-receptor pairs. Dot color indicated the mean interaction strength levels. **b**, Interaction strength/weights bewteen *STMN1*^*+*^*HMGN2*^*+*^*GPC3*^*+*^ cell subtype, other tumor cells and microenvironment cells. **c**, CypA signaling strength between the sender and receiver cells. **d**, Spatial plots of *PPIA* and *BSG* genes expression in P3 tissue. T, tumor region; PT, paratumor region; TC, tumor capsule region; and TR, transition state region. *PPIA*, Peptidylprolyl Isomerase A; *BSG*, Basigin. **e**, Immunofluorescence images of *PPIA* and *BSG* expression in MVI^−^ and MVI^+^ samples. **f and g**, The expression of *PPIA* (**f**) and *BSG* (**g**) in MVI^+^, MVI^−^ and different tumor grades, and their relationship with patient prognosis. NK, natural killer; OS, overall survival; HR, hazard ratio; MVI^−^, microvascular invasion negative; MVI^+^, microvascular invasion positive. Statistical analysis was performed using the Student *t* test (f and g).
(TIF)

S8 Fig TG biosynthesis decreased in *STMN1*+*HMGN2*+*GPC3*+ cell subtype. **a–c**, “triglyceride biosynthetic process” pathway activity comparison between MVI^+^ and MVI^−^ tumors in ST T region (**a**), TCGA-LIHC RNA-Seq (**b**) data and *STMN1*^*+*^*HMGN2*^*+*^*GPC3*^*+*^ cell subtype clusters (**c**). **d**, Manual region division of spatial metabolome H&E images (left) and metabolites intensity boxplot between regions of P1 and P4 (right).ST, spatial transcriptomics; PA, phosphatidic acid; DG, diacylglycerol; TG, triglyceride; T, tumor region; PT, paratumor region; TC, tumor capsule region; MVI^−^, microvascular invasion negative; MVI^+^, microvascular invasion positive; TCGA, the cancer genome atlas; LIHC, liver hepatocellular carcinoma. Statistical analysis was performed using the Student *t* test (a–c).
(TIF)

S9 Fig Spatial plots of CAFs subpopulations. **a**, Spatial plots of gene signature score for vCAF, mCAF, iCAFs and apCAFs in P1, P2 and P4 tissue (left) and spot distance to the tumor boundary (right). vCAF, vascular cancer-associated fibroblast; mCAF, myofibroblastic cancer-associated fibroblast; iCAF, inflammatory cancer-associated fibroblast; apCAF, antigen-presenting cancer-associated fibroblast; MVI^−^, microvascular invasion negative; MVI^+^, microvascular invasion positive. **b**, Spatial plots of gene signature scores for vCAF, mCAF, iCAFs and apCAFs in patient P3. **c**, Euclidean distance of spots in the TC region to the tumor boundary. T, tumor region; PT, paratumor region; TC, tumor capsule region; and TR, transition state region. **d**, Spearman correlation between CAFs signature scores and the distance of spots to the tumor boundary.
(TIF)

S10 Fig Taurine promotes HCC growth by suppressing iCAFs. **a**, Volcano plot showing the 166 differential metabolites (DMs) between the TC region and other regions. TC, tumor capsule sections; ns, no significance. **b**, Heatmap of hierarchical clustering of DMs across four tumor samples. **c**, Bar plot of KEGG pathways enriched in 36 DMs. **d**, Heatmap showing the Pearson correlation between 36 metabolites and cell proportions of the TC region. DEM, differential expression metabolites; NK, Natural killer. **e**, Heatmaps showing the mean of Pearson correlation between taurine (Ta) intensity and signature scores of four CAF subpopulations across four tumor samples. CAFs, cancer-associated fibroblasts; vCAFs, vascular CAFs; mCAFs, matrix CAFs; iCAFs, inflammatory CAFs; apCAFs, antigen-presenting CAFs. **f**, Effects of taurine on iCAF_HSC-NCCO_ proliferation evaluated using EDU assay. EDU, 5-ethynyl-2′-deoxyuridine; HSC, hepatic stellate cell; NCCO, non-contact co-culture. **g**, Transwell co-culture assay was used to detect the effect of iCAF_HSC-NCCO_ with or without taurine on tumor cell migration and invasion. Statistical analysis was performed using the Student *t* test (f and g).
(TIF)

S11 Fig Metabolites distribution and Schematic diagram of the image registration. **a**, Cluster distribution of Set 1 metabolites. **b**, spatial image of set 1 metabolite example. **c**, Cluster distribution of Set 2 metabolites. **d**, Schematic diagram of the image registration between ST and SM H&E images. ST, spatial transcriptomics; MSI, mass spectrometry imaging. SM, spatial metabolomics; H&E, hematoxylin and eosin staining; MVI^+^, microvascular invasion positive.
(TIF)

S12 Fig Spatial correlation between metabolite intensity and cell proportion. a–d, The heatmap demonstrates the Pearson correlation between the intensity of 36 metabolites of Set 2 and the proportion of cell types in P1 (a), P2 (b), P3 (c), and P4 (d) samples. NK, natural killer; MVI^−^, microvascular invasion negative, MVI+, Microvascular invasion positive.
(TIF)

S13 Fig TC region differential metabolites correlation with CAFs subpopulations in tumor samples. **a**, Heatmap of the mean of Pearson correlation between DEMs and CAFs subpopulation across 4 tumor samples. **b**, SM images of 4 CAFs related metabolites (C24H27ClN4O6, C7H9N2O, C4H5NO3S and C43H78NO7P) in all 6 samples. **c**, Taurine (C2H7NO3S) intensity distribution in SM images for all 6 samples. **d**, Pearson correlation between taurine intensity and the distance of spots to tumor boundary. DEM, differential expression metabolites; SM, spatial metabolomics; CAFS, cancer-associated fibroblasts; vCAF, vascular cancer-associated fibroblast; mCAF, myofibroblastic cancer-associated fibroblast; iCAF, inflammatory cancer-associated fibroblast; apCAF, antigen-presenting cancer-associated fibroblast; MVI^−^, microvascular invasion negative; MVI^+^, microvascular invasion positive.
(TIF)

S14 Fig Induction of the iCAF Phenotype. **a–c**, Immunofluorescence (**a**) and western blot (**b and c**) analyses confirmed the expression of α-SMA and collagen 1 (COL1) in mCAFs induced by *TGF-β*, and in iCAFs induced through non-contact co-culture (NCCO) with HSCs. **d**, qPCR detected the expression of iCAF biomarkers *IGF-1*, *CXCL2*, and *C7* following non-contact co-culture with HSCs. **e**, ELISA measured the concentrations of *IGF-1*, *CXCL2*, and *C7* in the supernatant, further confirming iCAF induction by HSC co-culture. **f and g**, qPCR and ELISA assays were performed to measure the expression levels of iCAF biomarkers *IGF-1*, *CXCL2*, and *C7* following NCCO induction of primary CAFs. α-SMA, α-smooth muscle actin; TGF-β, transforming growth factor beta; HSC, hepatic stellate cell; qPCR, quantitative polymerase chain reaction; IGF-1, insulin-like growth factor 1; CXCL2, C-X-C motif chemokine ligand 2; C7, complement component 7; ELISA, enzyme-linked immunosorbent assay; CAF, cancer-associated fibroblast; iCAF, inflammatory cancer-associated fibroblast; mCAF, myofibroblastic cancer-associated fibroblast; HSCs, hepatic stellate cells; GAPDH, glyceraldehyde-3-phosphate dehydrogenase; pCAF, primary cancer-associated fibroblast. Statistical analysis was performed using the Student *t* test (d–g).
(TIF)

S15 Fig Taurine suppressing the proliferation of iCAFs. **a**, The effects of taurine (Ta) on iCAF_HSC-NCCO_ proliferation were evaluated using CCK-8 assay. **b**, western blot analysis of the effect of taurine on *α-SMA* and *COL1* expression in iCAFs_HSC-NCCO_. **c and d**, qPCR (**c**), and ELISA assays (**d**) were utilized to determine the expression levels of iCAF markers *IGF-1*, *CXCL2*, and *C7* following taurine treatment. iCAF, inflammatory cancer-associated fibroblast; HSC, hepatic stellate cell; NCCO, non-contact co-culture; CCK-8, cell counting Kit-8; qPCR, quantitative polymerase chain reaction; *IGF-1*, insulin-like growth factor 1; *CXCL2*, C-X-C motif chemokine ligand 2; *C7*, complement component 7; ELISA, enzyme-linked immunosorbent assay; Ctrl, control; OD, optical density; COL1, collagen I; α-SMA, α-smooth muscle actin; GAPDH, glyceraldehyde-3-phosphate dehydrogenase. Statistical analysis was performed using the Student *t* test (a, c, and d).
(TIF)

S16 Fig Parallel verification that taurine does not affect tumor cell proliferation. **a–f**, The influence of taurine (Ta) on the proliferation of Huh7 (**a and b**), HepG2 (**c and d**), and Hep3B (**e and f**) tumor cells was assessed using EDU and CCK-8 assay. CCK-8: Cell Counting Kit-8, EDU: 5-ethynyl-2′-deoxyuridine, Ctrl, control. Statistical analysis was performed using the Student *t* test (a–f).
(TIF)

S17 Fig Primary CAFs verified that taurine promoted tumor cell migration and invasion by suppressing iCAFs. **a**, The impact of taurine (Ta) on iCAF_pCAF-NCCO_ proliferation was determined using the EDU assay. **b**, western blot analysis of the effect of taurine on *α-SMA* and *COL1* expression in iCAFs _pCAF-NCCO_. **c and d**, qPCR (**c**), and ELISA assays (**d**) confirmed the expression of *IGF-1*, *CXCL2*, and *C7* in iCAF_pCAF-NCCO_ after taurine treatment. **e**, Transwell co-culture assays examined the effect of iCAF_pCAF-NCCO_ on tumor cells migration and invasion with or without taurine. CAF, cancer-associated fibroblast; iCAF, inflammatory cancer-associated fibroblast; pCAF, primary cancer-associated fibroblast; NCCO, non-contact co-culture; EDU, 5-ethynyl-2′-deoxyuridine; qPCR, quantitative polymerase chain reaction; *IGF-1*, insulin-like growth factor 1; *CXCL2*, C-X-C motif chemokine ligand 2; *C7*, complement component 7; ELISA, enzyme-linked immunosorbent assay; COL1, collagen I; α-SMA, α-smooth muscle actin; GAPDH, glyceraldehyde-3-phosphate dehydrogenase. Statistical analysis was performed using the Student *t* test (a, c, d and e).
(TIF)

S1 Table Clinical information for each patient. N1 tissue is from patient P1. N3 tissue is from patient P3. MVI, microvascular invasion; HCC, hepatocellular carcinoma.
(XLSX)

S2 Table Exact *P* values corresponding to Fig 1g. (XLSX)

S3 Table Positive and negative ion metabolites annotation by *m*/*z.* ppm, part per million; HMDB, human metabolome database; KEGG, kyoto encyclopedia of genes and genomes; CAS, chemical abstracts service.
(XLSX)

S4 Table Positive ion metabolites clusters identified by spatial shrunken centroids clustering analysis. The shrunken centroid scores indicate the distinctive strength or representativeness of each metabolite within the corresponding cluster.
(XLSX)

S5 Table Differential gene expression analysis between MVI-positive and MVI-negative samples within the T region. Patient identity was used as a covariate in the linear equation for regional differentially expressed gene analysis.
(XLSX)

S6 Table KEGG pathway enrichment for DEGs of T region between HCC MVI+ and MVI^−^ tumors. (XLSX)

S7 Table DEGs of Scissor positive clusters SC_C6 and SC_C7 versus Scissor negative clusters in 79 scRNA-Seq malignant cells. (XLSX)

S8 Table Differential expression metabolites upregulated in the TC region. HMDB, human metabolome database; KEGG, kyoto encyclopedia of genes and genomes.
(XLSX)

S9 Table Spatial correlation between DEMs in TC region and cell type proportion. DEM, differential expression metabolites.
(XLSX)

S10 Table The gene-specific primers. (XLSX)

## Abbreviations

AFADESI-MSI: Airflow-Assisted Desorption Electrospray Ionization-Mass Spectrometry Imaging
AFP: alpha-fetoprotein
apCAFs: antigen-presenting CAFs
APOE⁺: apolipoprotein E positive
CAFs: cancer-associated fibroblasts
CCK8: cell counting kit-8
CI: confidence interval
CNV: copy number variations
CXCL14⁺: C-X-C Motif Chemokine Ligand 14 positive
DCN: decorin
DEGs: differentially expressed genes
DFS: disease-free survival
DG: diacylglycerols
EDU: 5-ethynyl-2′-deoxyuridine
FFPE: formalin-fixed, paraffin-embedded
GO: Gene Ontology
GSVA: Gene Set Variation Analysis
HCC: hepatocellular carcinoma
HMDB: Human Metabolome Database
HR: hazard ratio
HSCs: hepatic stellate cells
iCAFs: inflammatory cancer-associated fibroblasts
KEGG: Kyoto Encyclopedia of Genes and Genomes
LIHC: liver hepatocellular carcinoma
LPA: lysophosphatidic acid
mCAFs: matrix CAFs
MIF: multiplex immunofluorescence
MS: mass spectrometry
MVI: microvascular invasion
MVI^+^: MVI-positive
MVI^−^: MVI-negative
NK: natural killer
OS: overall survival
PA: phosphatidic acid
PCA: principal component analysis
ppm: parts per million
qPCR: quantitative real-time polymerase chain reaction
SA: Spatially Aware
SASA: Spatially Aware Structurally Adaptive
scRNA-seq: single-cell RNA sequencing
SM: spatial metabolomics
SSC: Spatial Shrunken Centroids
ST: spatial transcriptomics
TACE: transarterial chemoembolization
TG: triglycerides
TME: tumor microenvironment
UGs: upregulated genes
UMAP: Uniform Manifold Approximation and Projection
vCAFs: vascular CAFs
