# Supplementary material for: Spatial transcriptomic-metabolic features of tumor foci and tumor capsule in microvascular invasion with hepatocellular carcinoma: A spatial multi-omics study
Source: PLoS Med. 2026 May 15;23(5):e1004703. doi: 10.1371/journal.pmed.1004703 (PMC13178920; doi:10.1371/journal.pmed.1004703)
Supplement: S1 Raw Images — (PDF) [file pmed.1004703.s001.pdf]

Original images for blots and gels

"Spatial transcriptomic-metabolic features of tumor foci and tumor capsule in microvascular invasion with hepatocellular carcinoma: A spatial multi-omics study"

Zhi-Hui Luo [a, b, &], Na Wang [c, &], Jingwei Zhao [a, &], Fei Long [d], Si Wu [d], Wei Zhong [d], Wei-Ming Chen [a], Bicheng Wang [e], Kun Wang [f], Yufeng Yuan [g*], Jingjiao Zhou [a*], Chunhui Yuan [h, i*], Fubing Wang [b, h, j*]

[a]Department of Biology and Genetics, The College of Life Sciences and Health, Wuhan University of Science and Technology, Wuhan, Hubei, China.
[b]Center for Single-Cell Omics and Tumor Liquid Biopsy, Zhongnan Hospital of Wuhan University, Wuhan, Hubei, China.
[c]Department of Pathology, Renmin Hospital of Wuhan University, Wuhan, Hubei, China.
[d]Department of Laboratory Medicine, Zhongnan Hospital of Wuhan University, Wuhan, Hubei, China.
[e]Department of Pathology, Zhongnan Hospital of Wuhan University, Wuhan, Hubei, China
[f]Department of Laboratory Medicine, Hubei Cancer Hospital, Wuhan, Hubei, China.
[g]Department of Hepatobiliary and Pancreatic Surgery, Zhongnan Hospital of Wuhan University, Wuhan, Hubei, China.
[h]Department of Clinical Laboratory, Renmin Hospital of Wuhan University, Wuhan, Hubei, China.
[i]Institute of Clinical Molecular Diagnosis, Wuhan University, Wuhan, Hubei, China.
[j]Wuhan Research Center for Infectious Diseases and Cancer, Chinese Academy of Medical Sciences, Wuhan, Hubei, China.

[&]These authors have contributed equally to this work

[*] yuanyf1971@whu.edu.cn; zhoujj@wust.edu.cn; chunhuii.yuen@whu.edu.cn；wfb20042002@sina.com

Original gel image of S14b Fig .

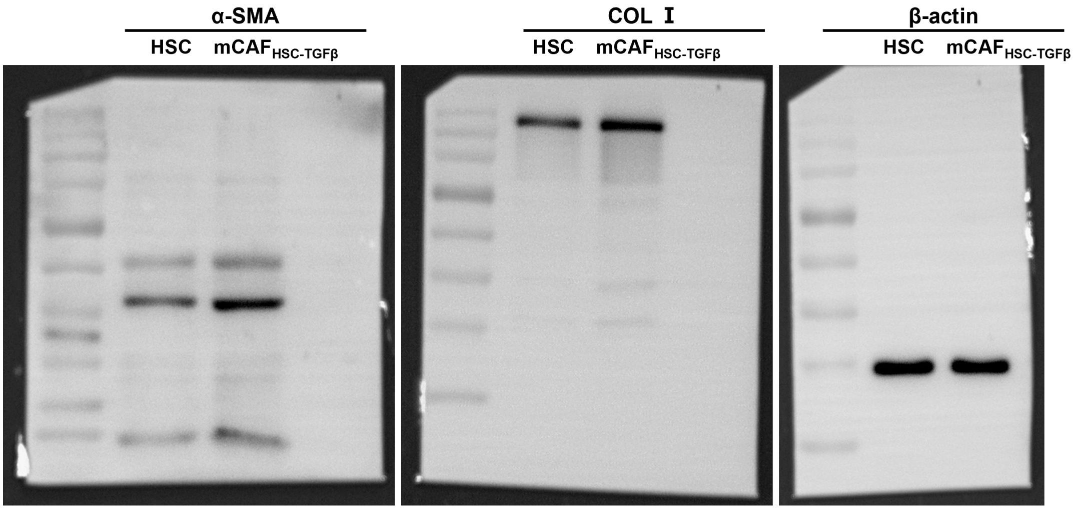

Original images for blots and gel of S14b Fig. Identification of α-SMA and COL1 expression in HSCs induced by TGF-β into mCAFs by Western blot. COL1: Collagen I, α-SMA: α-smooth muscle actin, TGF-β: Transforming growth factor beta, CAF: Cancer-associated fibroblast, mCAF: Myofibroblastic cancer-associated fibroblast, HSC: Hepatic stellate cell.

Original gel image of S14c Fig.

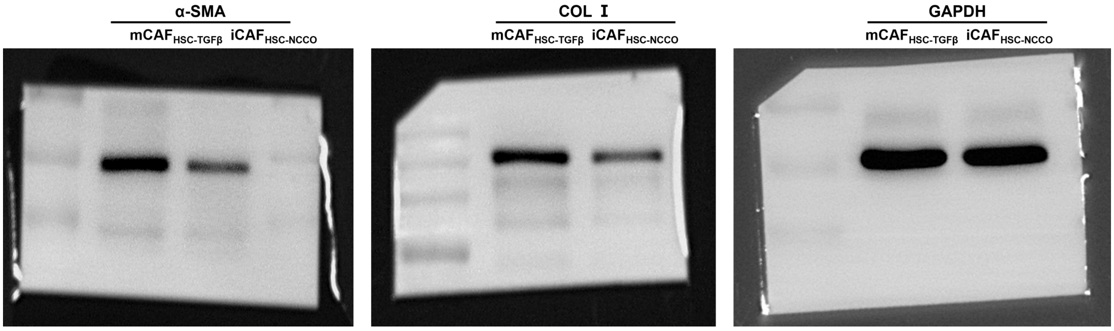

Original images for blots and gel of S14c Fig. Identification of α-SMA and COL1 expression in mCAFs induced by TGF-β, and in iCAFs induced through non-contact co-culture (NCCO) with HSCs by Western blot. COL1: Collagen I, α-SMA: α-smooth muscle actin, GAPDH: Glyceraldehyde-3-phosphate dehydrogenase, TGF-β: Transforming growth factor beta, CAF: Cancer-associated fibroblast, iCAF: Inflammatory cancer-associated fibroblast, mCAF: Myofibroblastic cancer-associated fibroblast, HSC: Hepatic stellate cell.

Original gel image of S15b Fig

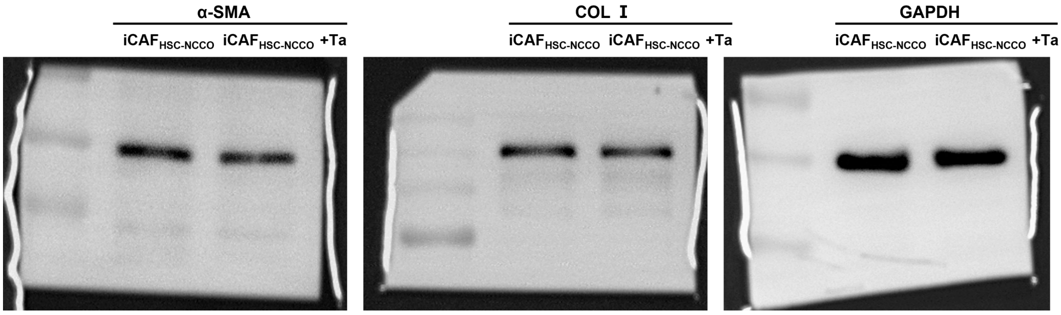

Original images for blots and gel of S15b Fig. Western blot analysis of the effect of taurine (Ta) on α-SMA and COL1 expression in iCAFs induced through non-contact co-culture (NCCO) with HSCs. COL1: Collagen I, α-SMA: α-smooth muscle actin, GAPDH: Glyceraldehyde-3-phosphate dehydrogenase, CAF: Cancer-associated fibroblast, iCAF: Inflammatory cancer-associated fibroblast, HSC: Hepatic stellate cell.

Original gel image of S17b Fig

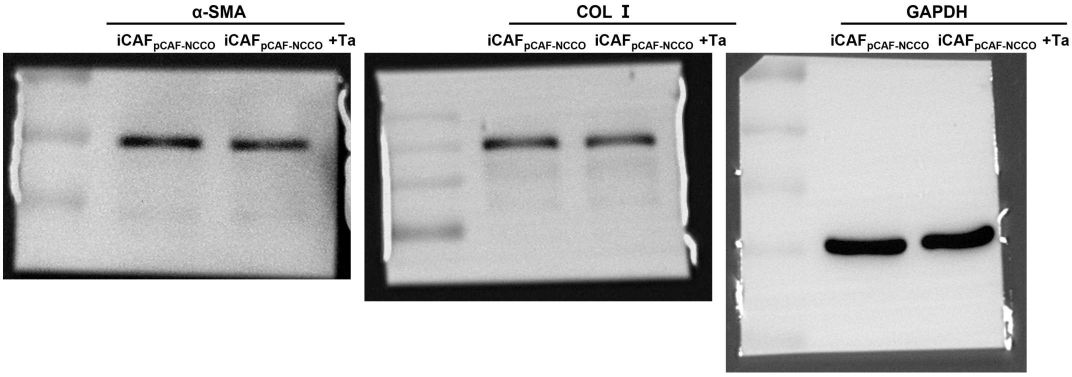

Original images for blots and gel of S17b Fig. Western blot analysis of the effect of taurine (Ta) on α-SMA and COL1 expression in iCAFs induced through non-contact co-culture (NCCO) with Primary CAFs (pCAFs). COL1: Collagen I, α-SMA: α-smooth muscle actin, GAPDH: Glyceraldehyde-3-phosphate dehydrogenase, CAF: Cancer-associated fibroblast, iCAF: Inflammatory cancer-associated fibroblast, pCAF: Primary cancer-associated fibroblast.
