# Supplementary material for: Spatial transcriptomic-metabolic features of tumor foci and tumor capsule in microvascular invasion with hepatocellular carcinoma: A spatial multi-omics study
Source: PLoS Med. 2026 May 15;23(5):e1004703. doi: 10.1371/journal.pmed.1004703 (PMC13178920; doi:10.1371/journal.pmed.1004703)
Supplement: S1 File — (DOCX) [file pmed.1004703.s002.docx]

**S1 File**

**“****Spatial transcriptomic-metabolic features of tumor foci and tumor capsule in microvascular invasion with hepatocellular carcinoma: A spatial multi-omics study”**

**Table of Contents**

**[Supplementary introduction](#_Toc222959478)** [1](#_Toc222959478)

**[Supplementary methods](#_Toc222959479)** [1](#_Toc222959479)

**[Supplementary results](#_Toc222959480)** [6](#_Toc222959480)

**[References](#_Toc222959481)** [9](#_Toc222959481)

# **Supplementary introduction**

**Analysis pipeline of this study**

In this study, we analyzed the tumor boundary tissues and distant peritumoral tissues from patients with Microvascular invasion positive (MVI^+^) and MVI negative (MVI^-^) Hepatocellular carcinoma (HCC) through spatial transcriptomics (ST) and spatial metabolomics (SM) profiling (**S1 Fig**). In the tumor region, the Stathmin 1 (STMN1)^+^ High Mobility Group Nucleosomal Binding Domain 2 (HMGN2)^+^ Glypican 3 (GPC3)^+^ cell subtype was identified as a MVI^+^ specific cellular population of HCC, which showed enhanced proliferative activity and metabolic reprogramming. Its prognostic value was further elucidated with online datasets and a local cohort of 79 patients. In the tumor capsule (TC) region, the spatial localization of inflammatory cancer-associated fibroblasts (iCAFs) distal to the tumor boundary was characterized, and taurine was identified as a cell-specific metabolite.

# **Supplementary methods**

**Copy number variation (CNV) inference**

We used inferCNV [1] to estimate CNVs for the 13 clusters of HCC tumor samples. Firstly, the spots of paratumor normal tissue N1 and N3 were combined as normal reference. Secondly, infercnv::run function was run on each tumor sample separately with the default parameters. The expression of CNV in segments was standardized and concentrated at 1. Fragment expression above 1 indicated CNV amplification, while fragment expression below 1 indicated CNV deletion. Thirdly, the CNV score of each spots was calculated with the following formula:

$\mathrm{CNV}_{i} = \frac{\sum_{j=1}^{n} {(c_{j} -1)}^{2}}{n}$ (1)

, where $\mathrm{CNV}_{i}$ is the CNV score of spot i; j is the segment in chromosome; n is the number of segments in chromosome.

**Cell type deconvolution**

The scRNA-Seq data of HCC were downloaded from GSE149614 [2]. Then, the scRNA-Seq data were classified into seven cell types, including hepatocytes, malignant hepatocytes (tumor), cancer associated fibroblasts (CAFs), endothelial cells, B cells, T/NK cells and myeloid cells. These cell populations were used as reference in RCTD [3] to deconvolute cell percentages in each spot. Cell percentages were compared between clusters with Wilcoxon test.

Based on the standardized cell proportions in each spot, hierarchical clustering was conducted for both rows (cell types) and columns (ST Clusters) using the Heatmap function. Concurrently, the Wilcoxon test was applied to compare cell percentages across different clusters. P values were adjusted by FDR.

**Cell interaction analysis**

Cell-cell interaction analysis between triple-positive malignant cells and other tumor clusters beyond microenvironment cells was performed by the CellChat package in the 79 scRNA-Seq data [4]. We used ligand-receptor (L-R) pairs of “Secreted Signaling” from CellChatDB. human as the background. We detected significant expressed L-R pairs using threshold values of log2 expression > 1 and P-value < 0.001. The interaction number and strength were summarized.

**Cell-type specific metabolites analysis**

Firstly, based on the H&E images of ST and spatial images of metabolism, we manually performed image registration through the software MSIReader. According to the spot positions of the ST, we found the corresponding spot area and position in the SM image, and then calculated the average metabolite intensity for spot in SM image as following steps.

Secondly, we extended the SM data and obtain a high resolution for it. We converted the spatial metabolic spot into pixels with an X-axis and Y-axis spacing of 100 um, and obtained the metabolite signal intensity of each pixel. Subsequently, we performed metabolite signal intensity estimation on the pixels between adjacent pixels (Formula 2). Next, we evaluated the metabolic signal intensity of intermediate pixels again using the new metabolic data obtained by the above method (Formula 3, 4). Then, we obtained the expanded spatial metabolic data with a resolution of 25 um. We chose all metabolism pixels located at the spatial transcriptome spot, and then estimated the metabolite ion intensity of that site based on the mean of these pixels. Finally, we obtained the one-to-one spot ion intensity for SM.

$\mathrm{Meta}_{i, {(a+b)}/2} = \frac{\mathrm{Meta}_{i, a} + \mathrm{Meta}_{i, b}}{2}$ (2)

$\mathrm{Meta}_{i, {(a +( a+b)}/2)/2} = \frac{\mathrm{Meta}_{i, a} + \mathrm{Meta}_{i, {(a+b)}/2}}{2}$ (3)

$\mathrm{Meta}_{i, {(b +( a+b)}/2)/2} = \frac{\mathrm{Meta}_{i, b} + \mathrm{Meta}_{i, {(a+b)}/2}}{2}$ (4)

, where the a and b are two adjacent spot in SM; i is a metabolite; $\mathrm{Meta}_{i, a}$ and $\mathrm{Meta}_{i, b}$ are metabolite intensity for i in position a and b; ${(a+b)}/2$ is the middle position between a and b; Finally, we can got three extended pseudo spots $\mathrm{Meta}_{i, {(a+b)}/2}$, $\mathrm{Meta}_{i, {(a +( a+b)}/2)/2}$ and $\mathrm{Meta}_{i, {(b +( a+b)}/2)/2}$ between spot a and spot b.

Thirdly, the obtained SM matrix has a one-to-one relationship with the ST matrix. Therefore, we created an assay using Seurat for the metabolite matrix and added it to the Seurat object of ST data. At the same time, we also created an assay for cell percentages and incorporate them into the aforementioned Seurat object. Based on this, we calculated the Pearson correlation between metabolites and cellular components, as well as metabolites and genes. We referred to this correlation as the spatial correlation of metabolites.

**Cell culture**

The human liver cancer cell lines (Huh7, HepG2 and Hep3B) and the HSC cell line LX2 were purchased from Cell Bank of Type Culture Collection of the Chinese Academy of Sciences (Shanghai Institute of Cell Biology) and maintained in DMEM (Gibco) supplemented with 10% FBS (Gibco) and 1% penicillin-streptomycin (Gibco). HSC cells were serum-starved for 24 h and subsequently treated with *TGF-β1* (10 ng/mL) for 24 h to generate activated HSC cells, which were designated as mCAFs. iCAFs were derived through a non-contact co-culture method [5]. All cell lines were cultured at 37°C in a humidified incubator with 5% CO₂.

**Isolation of fibroblasts**

Following the collection of fresh liver cancer tissues, samples were washed with PBS and finely chopped into pieces smaller than 1 mm³ using a surgical scalpel. The tissues were enzymatically dissociated in PBS containing 0.1% collagenase I at 37°C for 1 h. The dissociation was neutralized with complete culture medium (DMEM supplemented with 10% FBS and 1% penicillin-streptomycin), followed by centrifugation to remove the supernatant. The cell pellet was resuspended in complete culture medium and seeded into culture dishes for incubation. After 48 h, non-adherent cells and tissue debris were removed, and the adherent cells were washed twice with PBS. The fibroblasts were further incubated until they reached 80%-90% confluence and maintained at 37°C in a humidified incubator with 5% CO₂.

**Immunofluorescence**

Cells were fixed with 4% paraformaldehyde for 10 min to preserve cellular structures and permeabilized with 0.1% Triton X-100 for 15 min to facilitate antibody penetration. Blocking with 5% goat serum for 1 h at room temperature was performed to minimize non-specific binding. For tissue sections, deparaffinization was achieved using xylene for 10 min, followed by sequential rehydration through graded ethanol concentrations (100%, 95%, 80%, and 70%) and washing with distilled water. Antigen retrieval was carried out by heating the sections in citrate buffer (pH 6.0) at 95°C for 15 min. After cooling to room temperature, the sections were permeabilized with 0.3% Triton X-100 for 10 min and blocked with 5% goat serum for 1 h. Primary antibodies, including collagen I (Abcam, ab6308), α-SMA (Boster, BM0002), Anti-PPIA (Boster, A01308), and Anti-BSG (Boster, A00248-3), were incubated overnight at 4°C to ensure optimal antigen-antibody interactions. On the following day, the cells/sections were washed with PBS and incubated with secondary antibodies: FITC-conjugated (green) anti-rabbit IgG (Boster, BA1105) and Cy3-conjugated (red) anti-mouse IgG (Boster, BA1031) for 1 h in the dark to preserve fluorophore integrity. Nuclei were counterstained with DAPI for 10 min, and the samples were mounted with anti-fade medium to maintain fluorescence signals. Fluorescence signals were captured using a fluorescence microscope (NIKON ECLIPSE C1) for both cellular and tissue samples. Negative controls, in which primary antibodies were omitted, were included to confirm the specificity of the immunofluorescence staining and exclude non-specific background signals.

**Western blotting analysis**

The Western blotting assay was conducted following the standard protocol previously reported [6]. Cells were collected and lysed using RIPA buffer with a protease inhibitor cocktail (Beyotime, P0013C, China). Total protein concentrations were measured by the bicinchoninic acid (BCA) assay (Beyotime). Proteins were then immunoblotted with species-matched primary antibodies, including anti-GAPDH (Proteintech, 60004-1-Ig), anti-β-actin (Proteintech, 66009-1-Ig), anti-α-SMA (Proteintech, 67735-1-Ig), and anti-collagen I (abcam, ab6308), and incubated overnight at 4°C. Finally, the protein bands were visualized using an automatic chemiluminescence image analysis system (Bio-Rad, USA).

**RNA extraction and quantitative real-time polymerase chain reaction (qPCR)** **analysis**

Total RNA was extracted using the RNA isolation kit (Toyobo, Tokyo, Japan), following the manufacturer’s instructions. First-strand cDNA was synthesized using the Fermentas RevertAid First Strand cDNA Synthesis kit (Thermo Scientific). Quantitative real-time PCR was performed on an Applied Biosystems QuantStudio 5 Real-Time PCR System, using PerfeCTa qPCR FastMix (Quanta) and gene-specific primers ( **S10 Table**). GAPDH was used as an endogenous control. Relative mRNA expression levels were calculated using the 2^–ΔΔCt^ method.

**Cell counting kit-8 (CCK8) Assay**

Cells seeded into 96-well plates were incubated at 37°C for 0, 24, 48, and 72 h. Subsequently, 10 μL of CCK-8 (C10310-3, BS350B) mixed with 100 μL of complete culture medium was added to the cells for a 2-hour incubation. OD values were measured at 450 nm using a microplate reader.

**5-Ethynyl -2’- deoxyuridine (EDU) Assay**

Cell proliferation was assessed using an EDU kit (Ribobio, C10310-3). Cells in 96-well plates were incubated with 100 μL of EDU for 2 hours. They were then fixed with 4% paraformaldehyde for 15 min, treated with glycine (2 mg/mL) for 5 min, and permeabilized with 0.5% Triton-X100 for 10 min. The cells were stained with Apollo staining solution in the dark for 30 min, permeabilized again with 0.5% Triton-X100 for 10 min, and incubated with Hoechst for 30 min. Stained cells were imaged using a fluorescence microscope (Olympus, IX73).

**Co-culture, migration and invasion assays**

For the co-culture of CAFs and cancer cells, HCC cells were plated in the upper chamber of a transwell apparatus (0.8 μm insert; Corning, USA), and CAFs were cultured in the lower chamber. After incubation for 48 h, supernatants and the lower cell pellet were collected for further analysis. Cells that did not migrate through the upper chambers were removed with a cotton swab, fixed with 4% paraformaldehyde, and stained with crystal violet. Cells that migrated to the bottom side of membranes were counted under a microscope (Olympus, IX73). The invasion assay followed the same procedure as the migration assay, except that the upper chambers were precoated with Matrigel (1:8 dilution; BD Biosciences) at 37°C for 3 hours before cell inoculation.

**Enzyme-linked immunosorbent assay (ELISA)**

The co-culture medium was collected and used to assess the level of *IGF-1*, *CXCL2* and *C7* with Human *IGF-1* (Insulin-like Growth Factor 1) ELISA Kit (Elabscience, E-EL-H0086), Human *GROβ* (Growth Regulated Oncogene Beta)/*CXCL2* ELISA Kit (Elabscience, E-EL-H1904) and Human Complement Component 7 (*C7*) ELISA Kit (Jianglai Biology, JL12980-96T).

# **Supplementary results**

**iCAFs are located at distal to HCC tumor region**

We next determined whether the function of iCAFs is related to its specific spatial location. Given the spatial heterogeneity of cell states, we mapped the distribution of CAF subtypes using signatures for vascular CAFs (vCAFs), matrix CAFs (mCAFs), iCAFs, and antigen-presenting CAFs (apCAFs) [7]. Each CAF subtype exhibited distinct spatial localization relative to the tumor boundary (**S9a-c Fig**). The mCAF scores exhibited an inverse correlation with the distance from tumor boundaries (**S9d Fig)**, while the iCAF and apCAF signatures were intensified in distal regions. Together, these results suggested that iCAFs maintain specific spatial patterning in HCC architecture, and their functional attenuation is specifically associated with MVI^+^ status. The spatial distribution of iCAFs implied that examining the spatial distribution of metabolites could help identify those metabolites associated with iCAF function, thereby providing further insights into the role of iCAFs in tumor growth.

**Taurine is spatially correlated with iCAFs in HCC capsule region**

To identify the metabolites associated with MVI^+^, we first assessed differences in metabolite profiles within the TC region between MVI^+^ and MVI^−^, and then conducted a spatial correlation analysis to associate these metabolites with specific cell types, with a particular focus on the identification of iCAF-associated metabolites.

Differential metabolite (DM) intensity analysis identified a total of 166 metabolites, which were significantly (*P.adjust < 0.05*) upregulated in the TC region (**S10a Fig, S8 Table**). Clustering analysis of these DMs revealed two major sets of metabolites: Set 1 comprised 118 metabolites, and Set 2 included 36 metabolites (**S10b Fig**). Notably, the metabolites in Set 1 were preferentially localized to imaging artifacts at slide borders (**S11a-b Fig**), and were therefore excluded from downstream biological analyses. In contrast, the metabolites in Set 2 were primarily distributed within cluster SM_C11, with sparse distribution in clusters SM_C7, SM_C12, SM_C2, and SM_C6 **(S11c Fig)**. Pathway enrichment analysis of Set 2 metabolites identified “Taurine and hypotaurine metabolism” as the most significantly (*P* < 0.05) enriched metabolic pathway **(S10c Fig)**. These results demonstrated the major metabolites in the MVI^+^ TC region and their related functions.

To perform a spatial correlation analysis, we computationally integrated ST and SM data via multimodal image registration (**S11d Fig**). This integrative framework allows quantitative mapping of metabolite intensities to defined cellular niches within the ST dataset. The spatial correlation analysis revealed complex metabolic-cellular interaction networks (**S10d**, **S12 Fig**, **S9 Table**), with a subset of metabolites showing strong spatial co-localization with CAFs, such as C24H27ClN4O6 (clozapine glucuronide), C7H9N2O (1-methylnicotinamide), C4H5NO3S (2-oxothiazolidine-4-carboxylic acid), C2H7NO3S (taurine), C2H7NS (cysteamine), and C40H80NO8P (PC (32:0)) **(S10d Fig)**. Subtype-specific analysis revealed distinct metabolic dependencies, where C24H27ClN4O6 and C7H9N2O demonstrated stronger correlations with mCAFs, whereas C4H5NO3S, C43H78NO7P, and taurine showed preferential associations with iCAFs **(S13a-b Fig)**. Among these metabolites, taurine (C2H7NO3S) exhibited the most robust correlation with iCAFs (r = 0.38) compared with other CAF subtypes (r = [0.25, 0.33]) **(S10e, S13a Fig)**. Spatial metabolomics imaging further confirmed the enrichment of taurine within the TC region (**S13c Fig),** and its intensity was positively correlated with the proximity to iCAF-rich niches (**S13d Fig**). Collectively, these results indicated that taurine may participate in modulating iCAF activity within the tumor microenvironment (TME) of HCC.

**Taurine promotes HCC tumor growth by modulating iCAFs**

To investigate the functional relationship of taurine with iCAFs, hepatic stellate cells (HSCs) were first differentiated into iCAFs through non-contact co-culture (NCCO) with HCC cells [8]. NCCO-induced iCAFs demonstrated modest increases in *α-SMA* and collagen 1 (*COL1*) expression compared with baseline HSCs, while the expression was significantly (t-test, *P* < 0.001) lower than that in *TGF-β*-induced mCAFs (**S14a-c Fig**). Furthermore, three canonical iCAF markers (*IGF-1*, *CXCL2*, and *C7*) were detected to validate the induction of iCAFs [7, 9, 10]. NCCO-treated HSCs exhibited substantial upregulation of these markers at the transcriptional and secretory levels, confirming successful iCAF differentiation (**S14d-e Fig**). In addition, the induction of iCAFs was further replicated in primary CAFs isolated from human HCC specimens (**S14f-g Fig**). The addition of taurine significantly (t-test, *P* < 0.001) inhibited the proliferation of iCAFs **(Figs 7f, S15a**), along with decreases in *IGF-1*, *CXCL2*, and C7 expression (**S15b-d Fig**). In contrast, taurine exposure showed no direct anti-proliferative effect on the three distinct HCC cell lines, including Huh7, HepG2, and Hep3B (**S16a-f Fig**). Interestingly, the migration and invasion of HCC cells were markedly suppressed by iCAF in iCAF-tumor co-culture systems, which was reversed by the addition of taurine (**S10g Fig**). These findings were corroborated in primary CAF models (**S17a-e Fig**). Collectively, taurine may modulate tumor progression indirectly through iCAF functional reprogramming rather than directly through targeting HCC cells.

# **References**

1. Patel AP, Tirosh I, Trombetta JJ, Shalek AK, Gillespie SM, Wakimoto H, et al. Single-cell RNA-seq highlights intratumoral heterogeneity in primary glioblastoma. Science (New York, NY). 2014;344(6190):1396-401.doi: 10.1126/science.1254257. PMID: 24925914.

2. Lu Y, Yang A, Quan C, Pan Y, Zhang H, Li Y, et al. A single-cell atlas of the multicellular ecosystem of primary and metastatic hepatocellular carcinoma. Nat Commun. 2022;13(1):4594.doi: 10.1038/s41467-022-32283-3. PMID: 35933472.

3. Cable DM, Murray E, Zou LS, Goeva A, Macosko EZ, Chen F, et al. Robust decomposition of cell type mixtures in spatial transcriptomics. Nat Biotechnol. 2022;40(4):517-26.doi: 10.1038/s41587-021-00830-w. PMID: 33603203.

4. Jin S, Guerrero-Juarez CF, Zhang L, Chang I, Ramos R, Kuan CH, et al. Inference and analysis of cell-cell communication using CellChat. Nat Commun. 2021;12(1):1088.doi: 10.1038/s41467-021-21246-9. PMID: 33597522.

5. Öhlund D, Handly-Santana A, Biffi G, Elyada E, Almeida AS, Ponz-Sarvise M, et al. Distinct populations of inflammatory fibroblasts and myofibroblasts in pancreatic cancer. J Exp Med. 2017;214(3):579-96.doi: 10.1084/jem.20162024. PMID: 28232471.

6. Wang S, Qu Y, Fang X, Ding Q, Zhao H, Yu X, et al. Decorin: a potential therapeutic candidate for ligamentum flavum hypertrophy by antagonizing TGF-β1. Exp Mol Med. 2023;55(7):1413-23.doi: 10.1038/s12276-023-01023-y. PMID: 37394592.

7. Cords L, Tietscher S, Anzeneder T, Langwieder C, Rees M, de Souza N, et al. Cancer-associated fibroblast classification in single-cell and spatial proteomics data. Nat Commun. 2023;14(1):4294.doi: 10.1038/s41467-023-39762-1. PMID: 37463917.

8. Han C, Liu T, Yin R. Biomarkers for cancer-associated fibroblasts. Biomark Res. 2020;8(1):64.doi: 10.1186/s40364-020-00245-w. PMID: 33292666.

9. Chhabra Y, Weeraratna AT. Fibroblasts in cancer: Unity in heterogeneity. Cell. 2023;186(8):1580-609.doi: 10.1016/j.cell.2023.03.016. PMID: 37059066.

10. Zhang M, Yang H, Wan L, Wang Z, Wang H, Ge C, et al. Single-cell transcriptomic architecture and intercellular crosstalk of human intrahepatic cholangiocarcinoma. J Hepatol. 2020;73(5):1118-30.doi: 10.1016/j.jhep.2020.05.039. PMID: 32505533.
