# Supplementary figures and images for: Spatial transcriptomic-metabolic features of tumor foci and tumor capsule in microvascular invasion with hepatocellular carcinoma: A spatial multi-omics study
Source: PLoS Med. 2026 May 15;23(5):e1004703. doi: 10.1371/journal.pmed.1004703 (PMC13178920; doi:10.1371/journal.pmed.1004703)

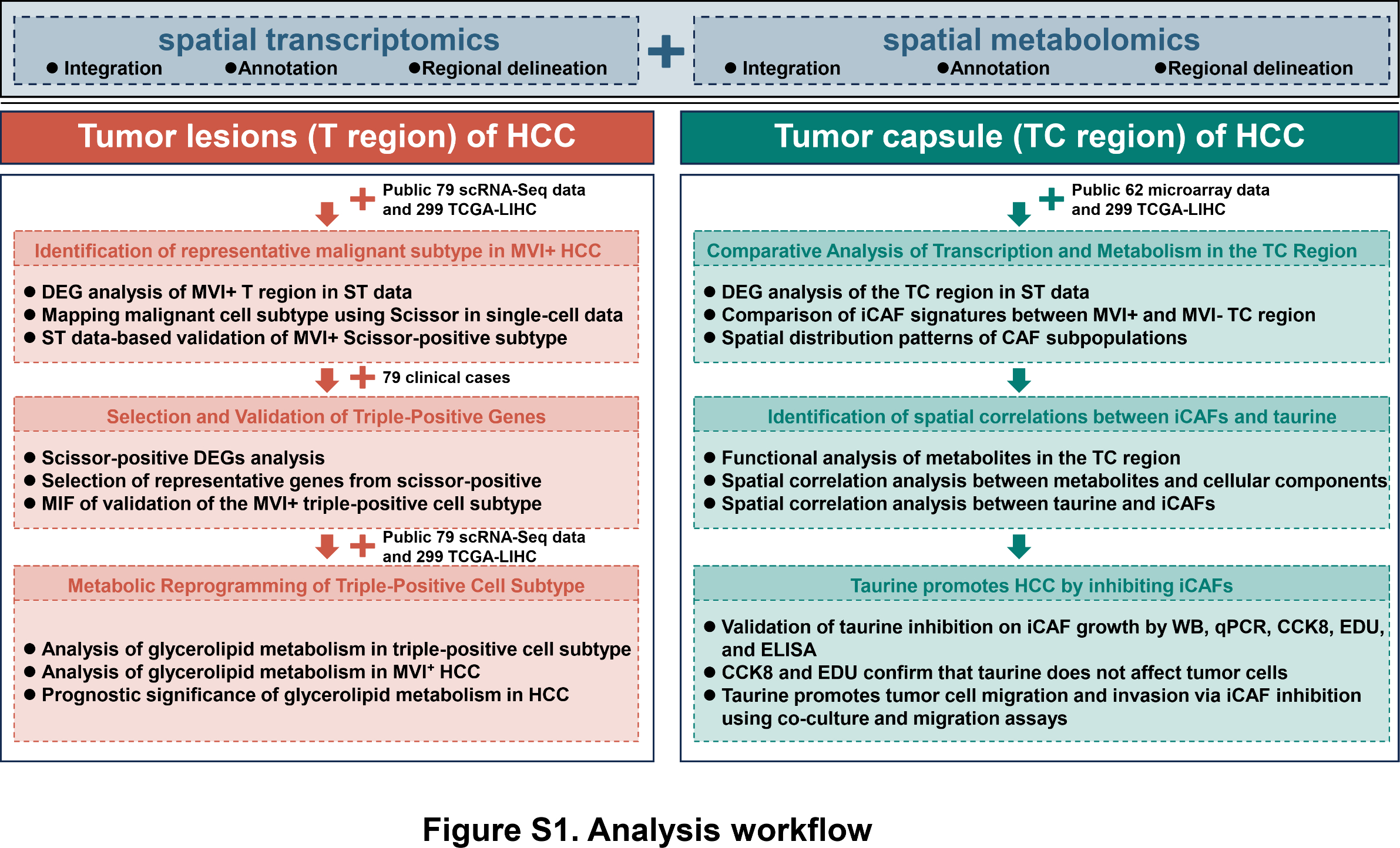

Supplement: S1 Fig — ST, spatial transcriptomics; DEG, differentially expressed gene; MIF, multiplex immunofluorescence; WB, western blot; qPCR, quantitative real-time polymerase chain reaction; CCK8, cell counting kit-8; EDU, 5-ethynyl −2′- deoxyuridine; TCGA, the cancer genome atlas; LIHC, liver hepatocellular carcinoma; MVI+, microvascular invasion positive; MVI−, microvascular invasion negative; TC, tumor capsule region; CAF, cancer-associated fibroblast; iCAF, inflammatory cancer-associated fibroblast; qPCR, quantitative polymerase chain reaction; ELISA, enzyme-linked immunosorbent assay; HCC, hepatocellular carcinoma; T, tumor; scRNA-seq, single-cell RNA sequencing. (TIF) [file pmed.1004703.s003.tif]

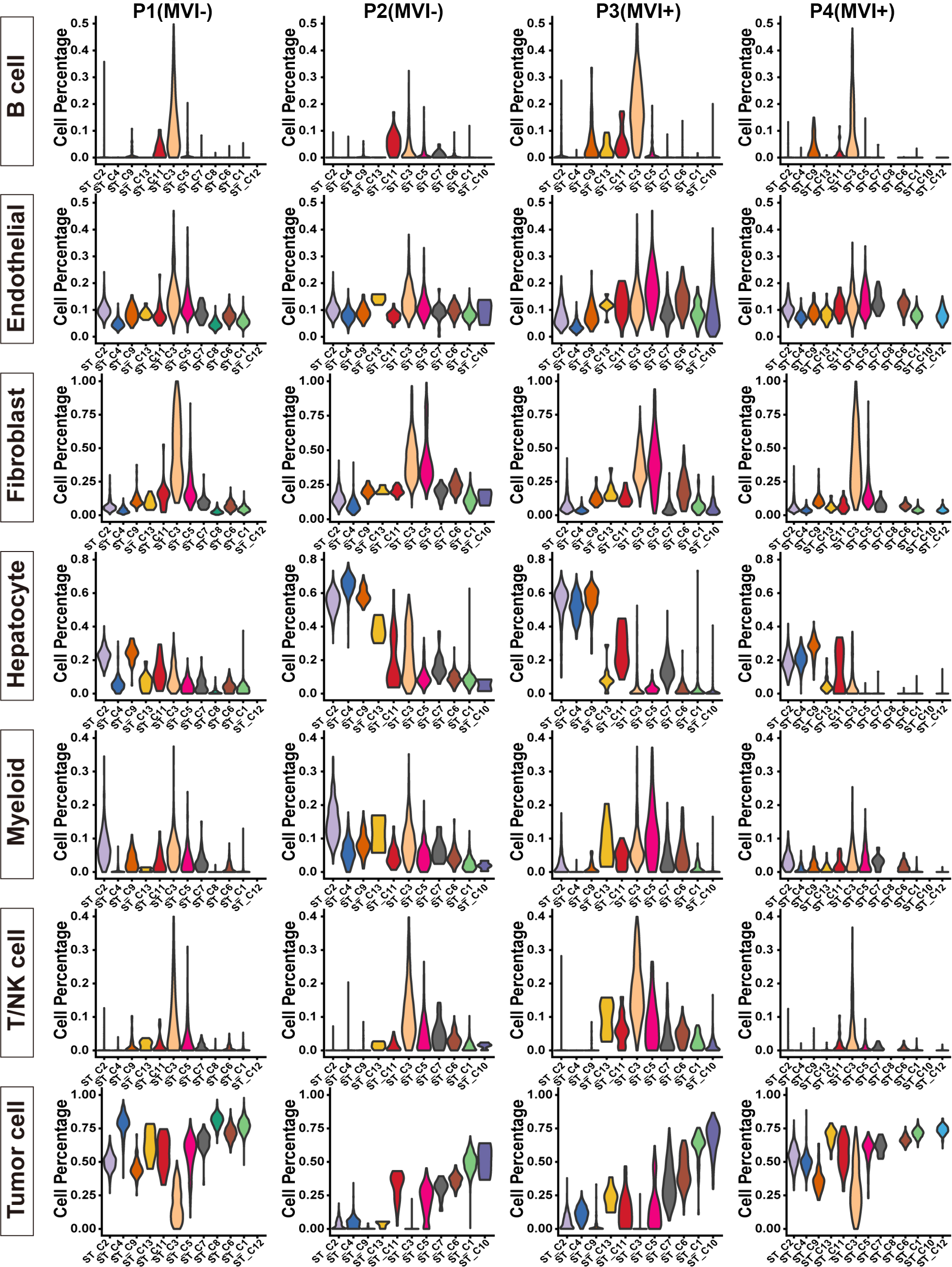

Supplement: S3 Fig — The violin plots showing cells (hepatocytes, malignant hepatocytes (tumor), Fibroblasts, endothelial cells, B cells, T/NK cells and myeloid cells) proportion in the integrated clusters for each sample. NK, Natural killer; ST, spatial transcriptomics. (TIF) [file pmed.1004703.s005.tif]

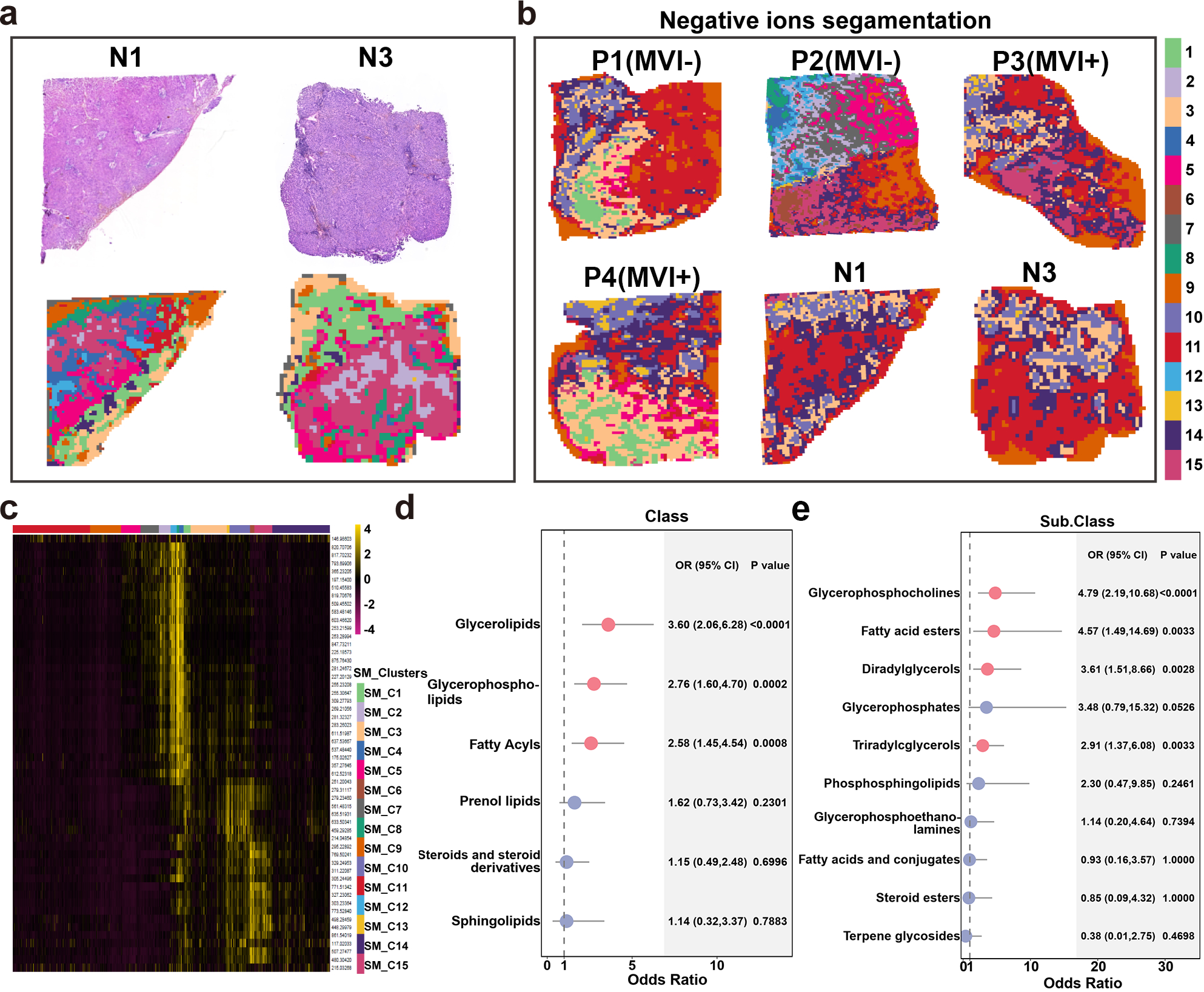

Supplement: S5 Fig — a, Overview of spatial metabolism clusters for positive ions in N1 and N3 samples. b, Overview of spatial metabolism clusters for negative ions in each sample. c, Heatmap plot showing top 10 negative ions intensity in each cluster. d and e, The odds ratio of metabolites counts in tumor region (clusters SM_C2, SM_C10, SM_C13 and SM_C15) over that in the whole tissue by class (d) and sub class (e). Red dots represent a P-value of Fisher’s test <0.05, while blue dots represent a P-value of Fisher’s test >0.05. SM, spatial metabolomics; OR, odds ratio; CI, confidence interval; MVI+, microvascular invasion positive; MVI−, microvascular invasion negative. (TIF) [file pmed.1004703.s007.tif]

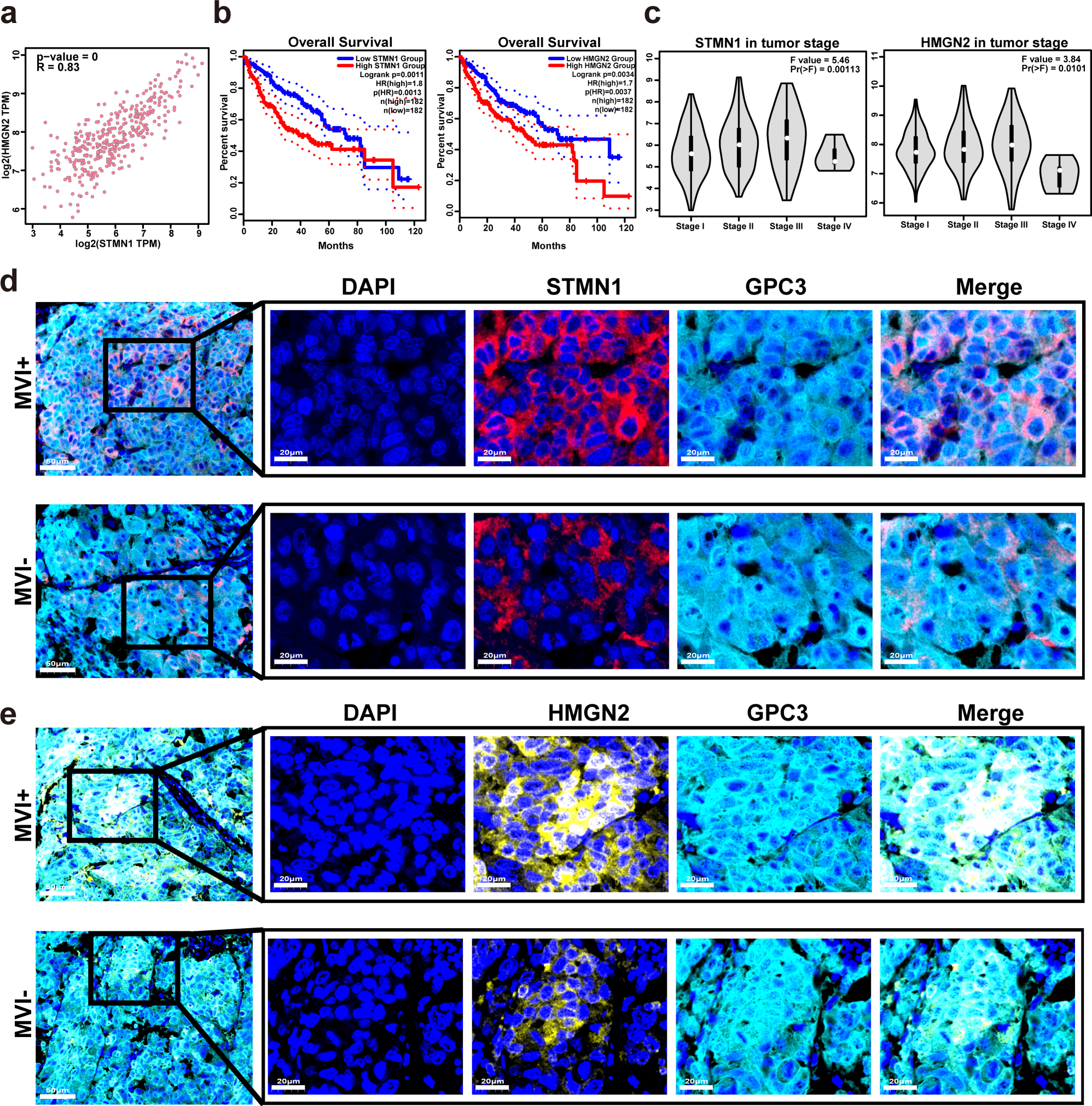

Supplement: S6 Fig — a, Correlation between STMN1 and HMGN2 genes. TPM, transcripts per million. b, Overall survival of STMN1 and HMGN2 genes score in HCC cohort. c, Violin plot of STMN1 and HMGN2 genes expression scores of tumor stages, d and e, Multiplex immunofluorescence of STMN1+GPC3+ (d) and HMGN2+GPC3+ (e) cells in MVI+ and MVI− samples. HR, hazard ratio; MVI−, microvascular invasion negative; MVI+, microvascular invasion positive. (TIF) [file pmed.1004703.s008.tif]

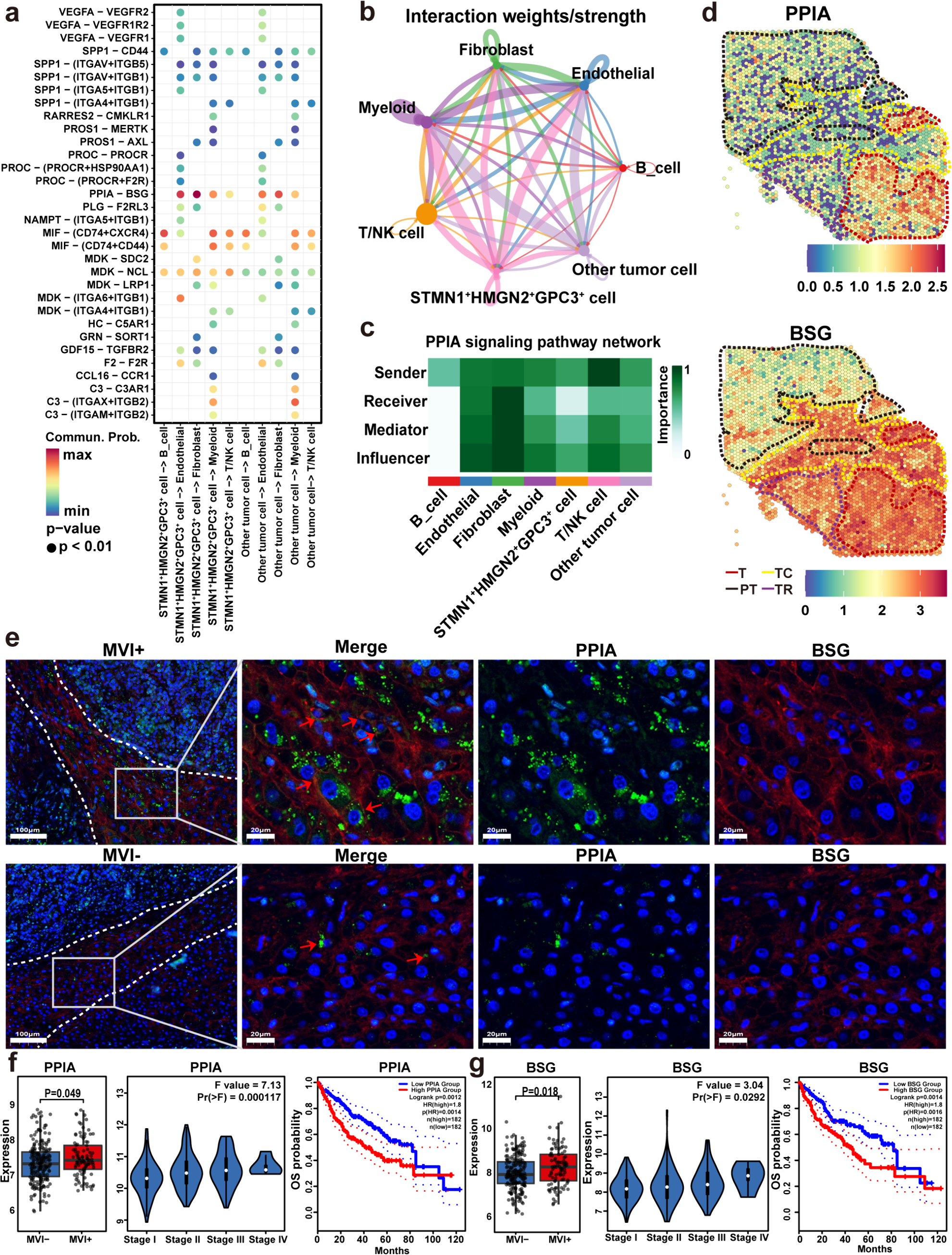

Supplement: S7 Fig — a, Bubble Heatmap showing the mean interaction strength between STMN1+HMGN2+GPC3+ cell subtype and other tumor cells for ligand-receptor pairs. Dot color indicated the mean interaction strength levels. b, Interaction strength/weights bewteen STMN1+HMGN2+GPC3+ cell subtype, other tumor cells and microenvironment cells. c, CypA signaling strength between the sender and receiver cells. d, Spatial plots of PPIA and BSG genes expression in P3 tissue. T, tumor region; PT, paratumor region; TC, tumor capsule region; and TR, transition state region. PPIA, Peptidylprolyl Isomerase A; BSG, Basigin. e, Immunofluorescence images of PPIA and BSG expression in MVI− and MVI+ samples. f and g, The expression of PPIA (f) and BSG (g) in MVI+, MVI− and different tumor grades, and their relationship with patient prognosis. NK, natural killer; OS, overall survival; HR, hazard ratio; MVI−, microvascular invasion negative; MVI+, microvascular invasion positive. Statistical analysis was performed using the Student t test (f and g). (TIF) [file pmed.1004703.s009.tif]

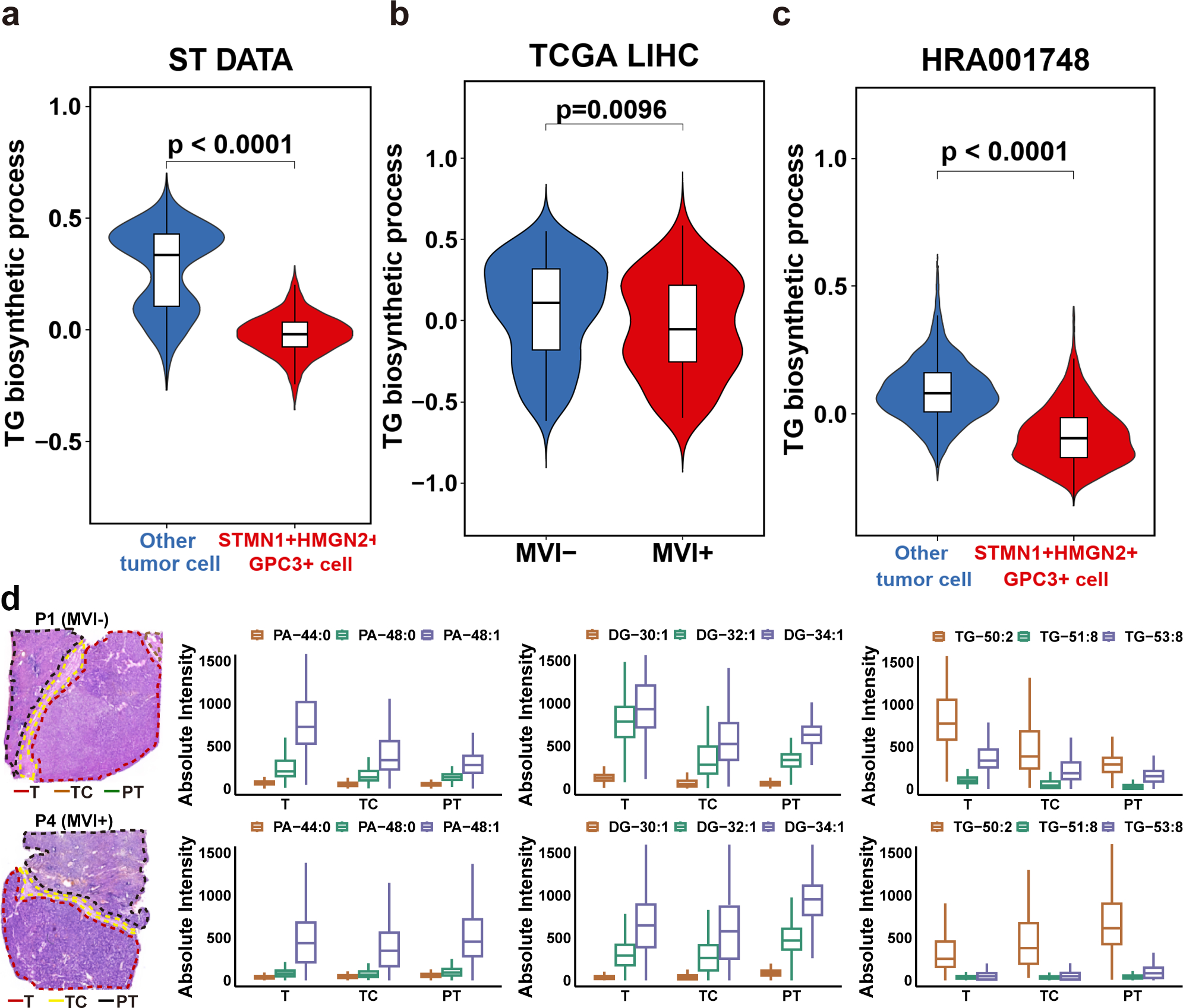

Supplement: S8 Fig — a–c, “triglyceride biosynthetic process” pathway activity comparison between MVI+ and MVI− tumors in ST T region (a), TCGA-LIHC RNA-Seq (b) data and STMN1+HMGN2+GPC3+ cell subtype clusters (c). d, Manual region division of spatial metabolome H&E images (left) and metabolites intensity boxplot between regions of P1 and P4 (right).ST, spatial transcriptomics; PA, phosphatidic acid; DG, diacylglycerol; TG, triglyceride; T, tumor region; PT, paratumor region; TC, tumor capsule region; MVI−, microvascular invasion negative; MVI+, microvascular invasion positive; TCGA, the cancer genome atlas; LIHC, liver hepatocellular carcinoma. Statistical analysis was performed using the Student t test (a–c). (TIF) [file pmed.1004703.s010.tif]

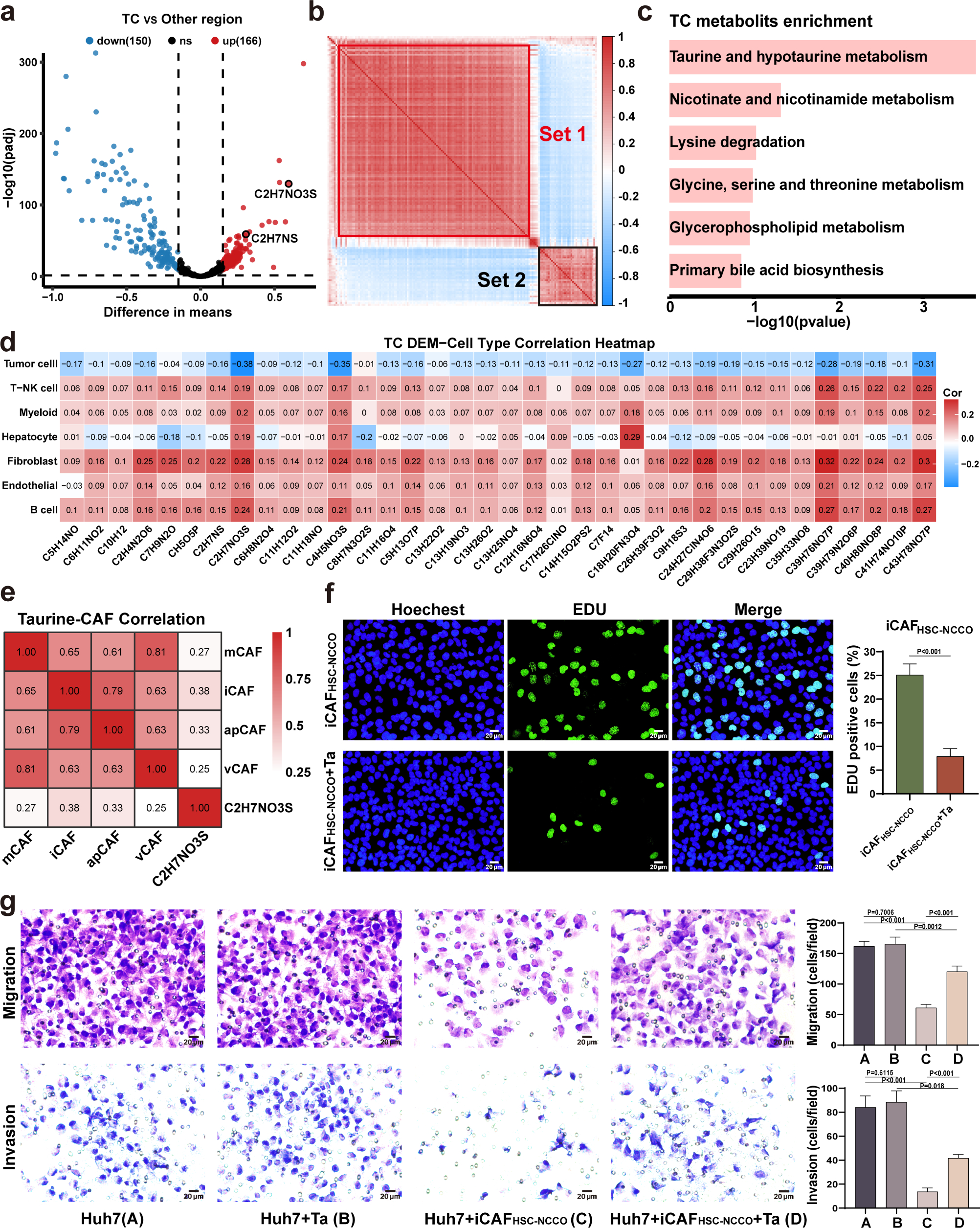

Supplement: S10 Fig — a, Volcano plot showing the 166 differential metabolites (DMs) between the TC region and other regions. TC, tumor capsule sections; ns, no significance. b, Heatmap of hierarchical clustering of DMs across four tumor samples. c, Bar plot of KEGG pathways enriched in 36 DMs. d, Heatmap showing the Pearson correlation between 36 metabolites and cell proportions of the TC region. DEM, differential expression metabolites; NK, Natural killer. e, Heatmaps showing the mean of Pearson correlation between taurine (Ta) intensity and signature scores of four CAF subpopulations across four tumor samples. CAFs, cancer-associated fibroblasts; vCAFs, vascular CAFs; mCAFs, matrix CAFs; iCAFs, inflammatory CAFs; apCAFs, antigen-presenting CAFs. f, Effects of taurine on iCAFHSC-NCCO proliferation evaluated using EDU assay. EDU, 5-ethynyl-2′-deoxyuridine; HSC, hepatic stellate cell; NCCO, non-contact co-culture. g, Transwell co-culture assay was used to detect the effect of iCAFHSC-NCCO with or without taurine on tumor cell migration and invasion. Statistical analysis was performed using the Student t test (f and g). (TIF) [file pmed.1004703.s012.tif]

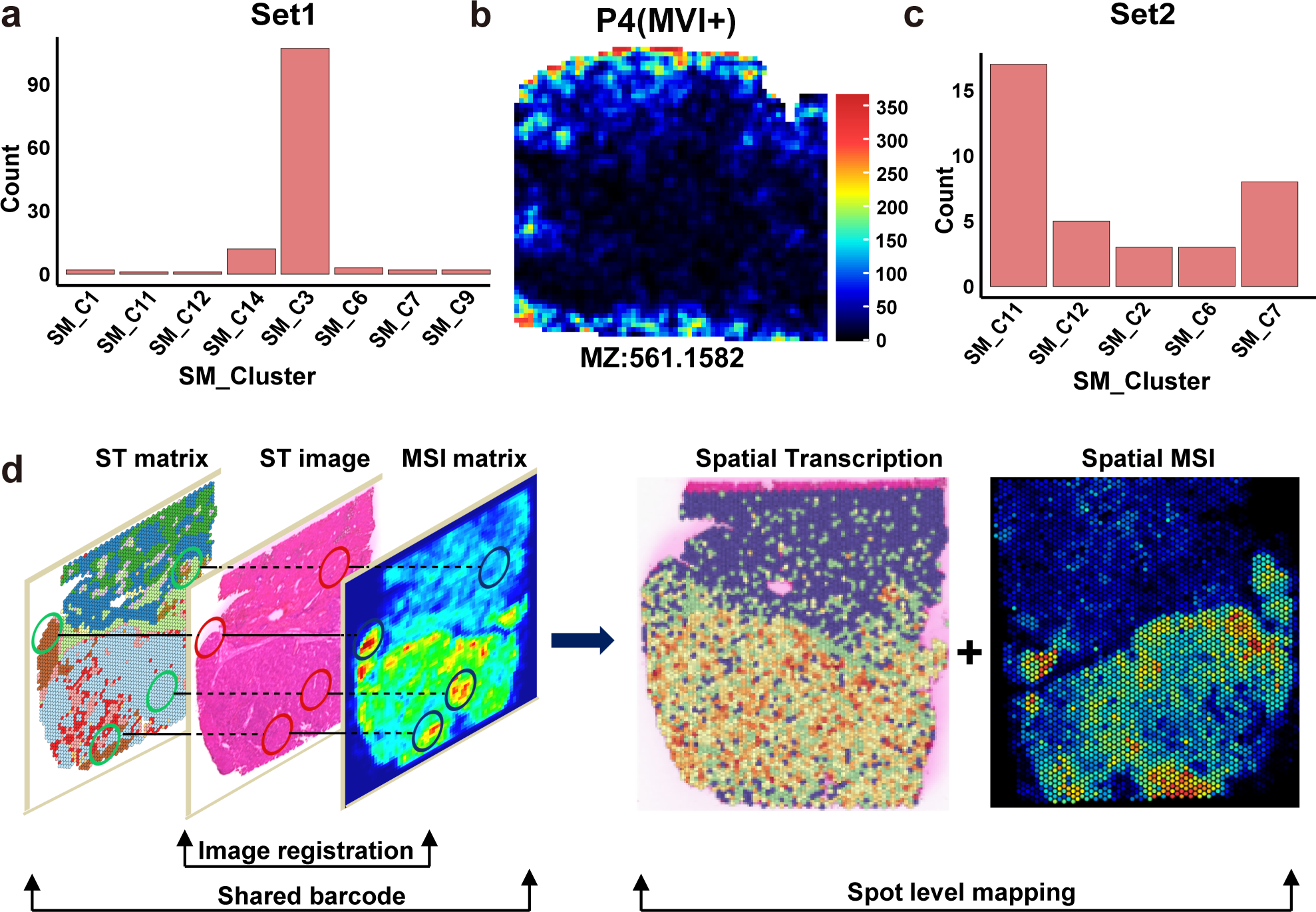

Supplement: S11 Fig — a, Cluster distribution of Set 1 metabolites. b, spatial image of set 1 metabolite example. c, Cluster distribution of Set 2 metabolites. d, Schematic diagram of the image registration between ST and SM H&E images. ST, spatial transcriptomics; MSI, mass spectrometry imaging. SM, spatial metabolomics; H&E, hematoxylin and eosin staining; MVI+, microvascular invasion positive. (TIF) [file pmed.1004703.s013.tif]

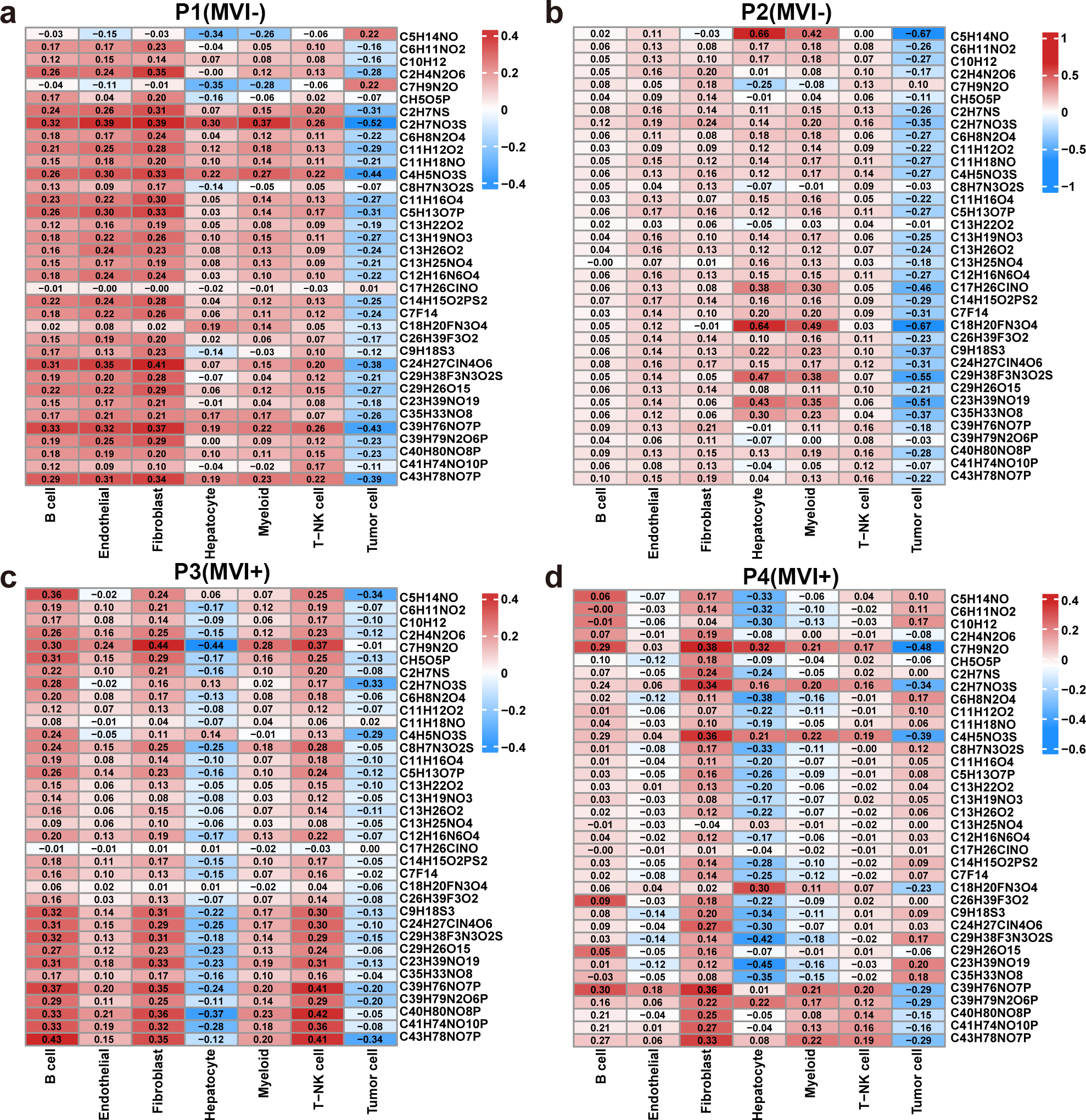

Supplement: S12 Fig — a–d, The heatmap demonstrates the Pearson correlation between the intensity of 36 metabolites of Set 2 and the proportion of cell types in P1 (a), P2 (b), P3 (c), and P4 (d) samples. NK, natural killer; MVI−, microvascular invasion negative, MVI+, Microvascular invasion positive. (TIF) [file pmed.1004703.s014.tif]

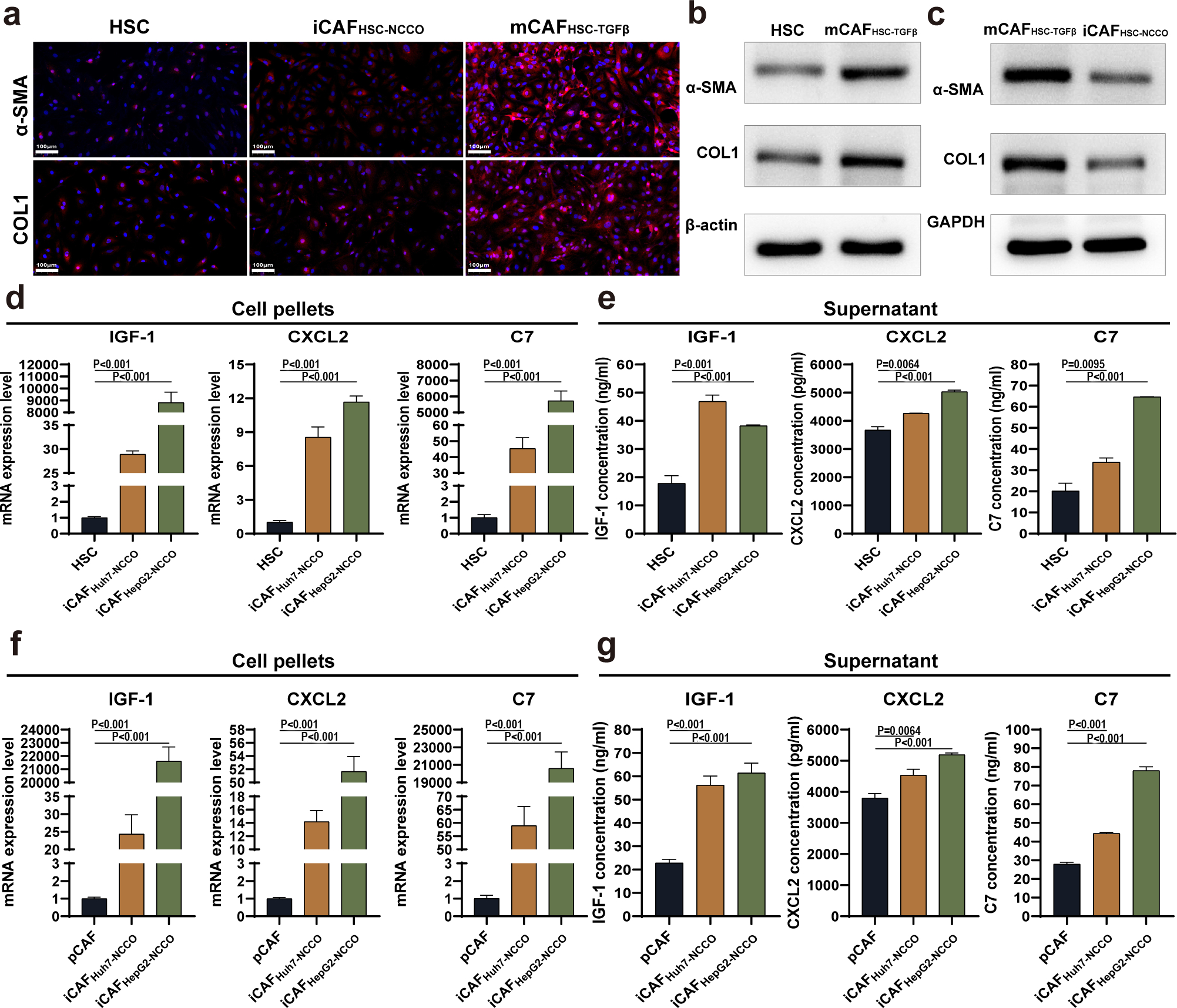

Supplement: S14 Fig — a–c, Immunofluorescence (a) and western blot (b and c) analyses confirmed the expression of α-SMA and collagen 1 (COL1) in mCAFs induced by TGF-β, and in iCAFs induced through non-contact co-culture (NCCO) with HSCs. d, qPCR detected the expression of iCAF biomarkers IGF-1, CXCL2, and C7 following non-contact co-culture with HSCs. e, ELISA measured the concentrations of IGF-1, CXCL2, and C7 in the supernatant, further confirming iCAF induction by HSC co-culture. f and g, qPCR and ELISA assays were performed to measure the expression levels of iCAF biomarkers IGF-1, CXCL2, and C7 following NCCO induction of primary CAFs. α-SMA, α-smooth muscle actin; TGF-β, transforming growth factor beta; HSC, hepatic stellate cell; qPCR, quantitative polymerase chain reaction; IGF-1, insulin-like growth factor 1; CXCL2, C-X-C motif chemokine ligand 2; C7, complement component 7; ELISA, enzyme-linked immunosorbent assay; CAF, cancer-associated fibroblast; iCAF, inflammatory cancer-associated fibroblast; mCAF, myofibroblastic cancer-associated fibroblast; HSCs, hepatic stellate cells; GAPDH, glyceraldehyde-3-phosphate dehydrogenase; pCAF, primary cancer-associated fibroblast. Statistical analysis was performed using the Student t test (d–g). (TIF) [file pmed.1004703.s016.tif]

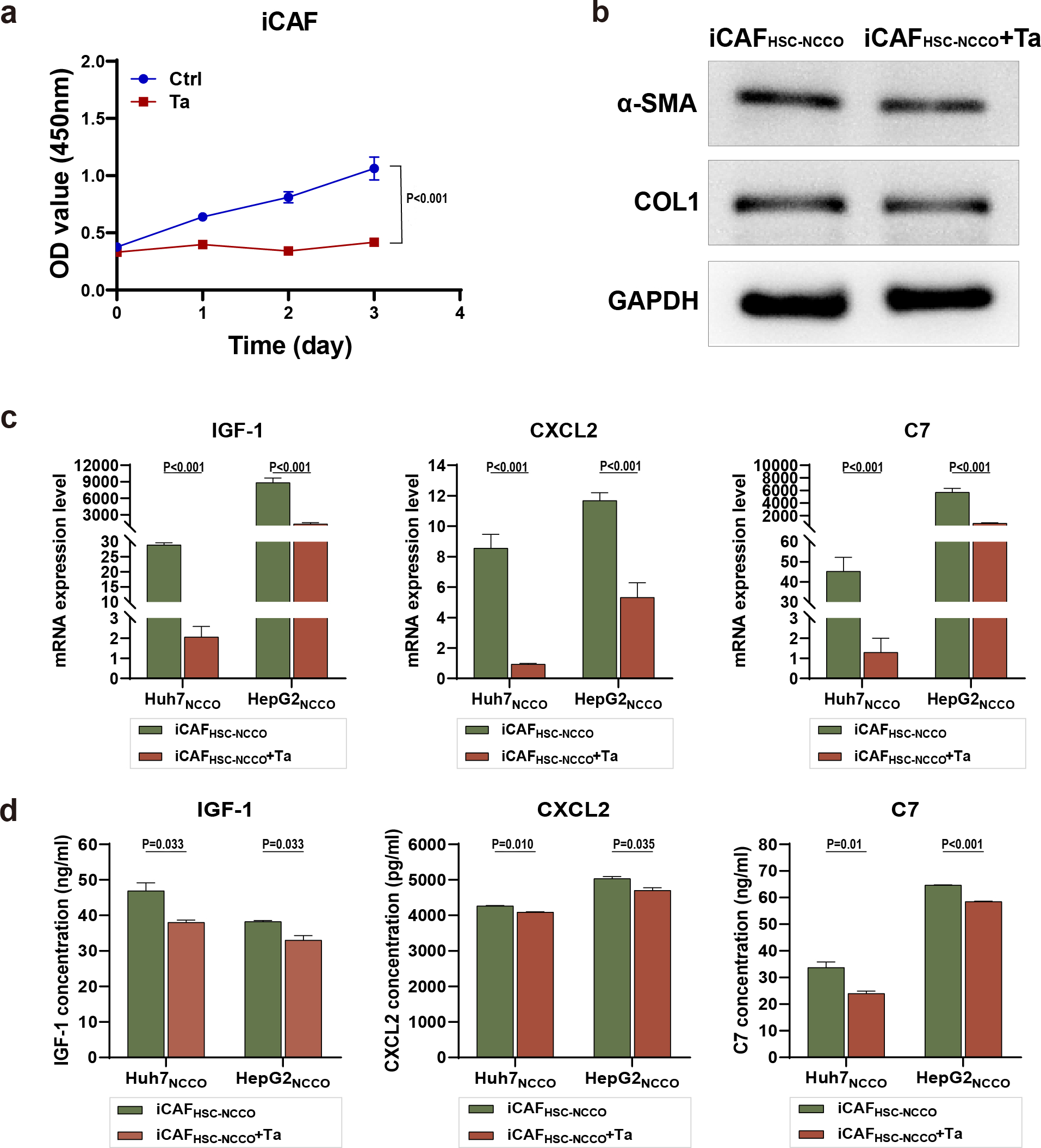

Supplement: S15 Fig — a, The effects of taurine (Ta) on iCAFHSC-NCCO proliferation were evaluated using CCK-8 assay. b, western blot analysis of the effect of taurine on α-SMA and COL1 expression in iCAFsHSC-NCCO. c and d, qPCR (c), and ELISA assays (d) were utilized to determine the expression levels of iCAF markers IGF-1, CXCL2, and C7 following taurine treatment. iCAF, inflammatory cancer-associated fibroblast; HSC, hepatic stellate cell; NCCO, non-contact co-culture; CCK-8, cell counting Kit-8; qPCR, quantitative polymerase chain reaction; IGF-1, insulin-like growth factor 1; CXCL2, C-X-C motif chemokine ligand 2; C7, complement component 7; ELISA, enzyme-linked immunosorbent assay; Ctrl, control; OD, optical density; COL1, collagen I; α-SMA, α-smooth muscle actin; GAPDH, glyceraldehyde-3-phosphate dehydrogenase. Statistical analysis was performed using the Student t test (a, c, and d). (TIF) [file pmed.1004703.s017.tif]

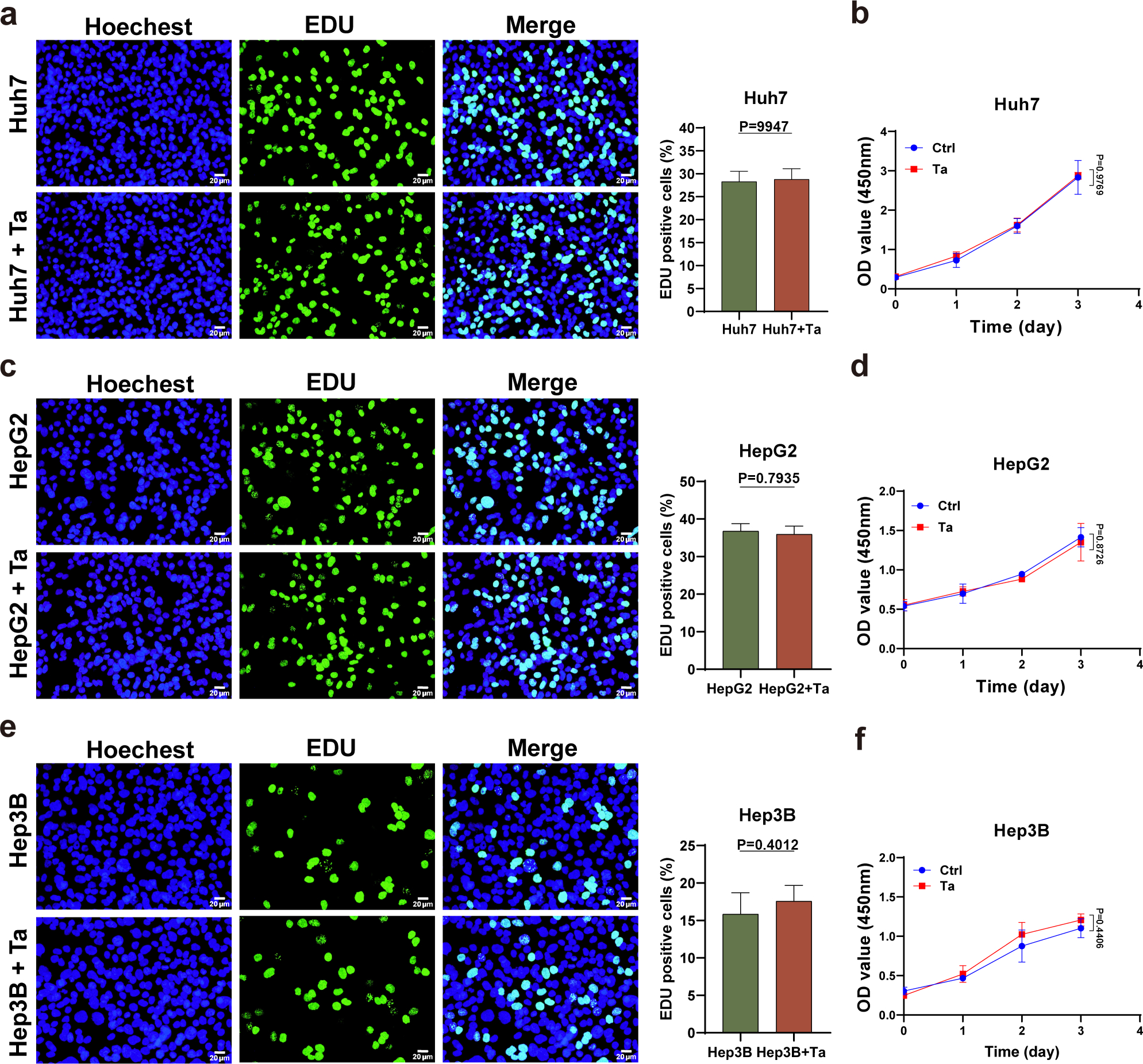

Supplement: S16 Fig — a–f, The influence of taurine (Ta) on the proliferation of Huh7 (a and b), HepG2 (c and d), and Hep3B (e and f) tumor cells was assessed using EDU and CCK-8 assay. CCK-8: Cell Counting Kit-8, EDU: 5-ethynyl-2′-deoxyuridine, Ctrl, control. Statistical analysis was performed using the Student t test (a–f). (TIF) [file pmed.1004703.s018.tif]

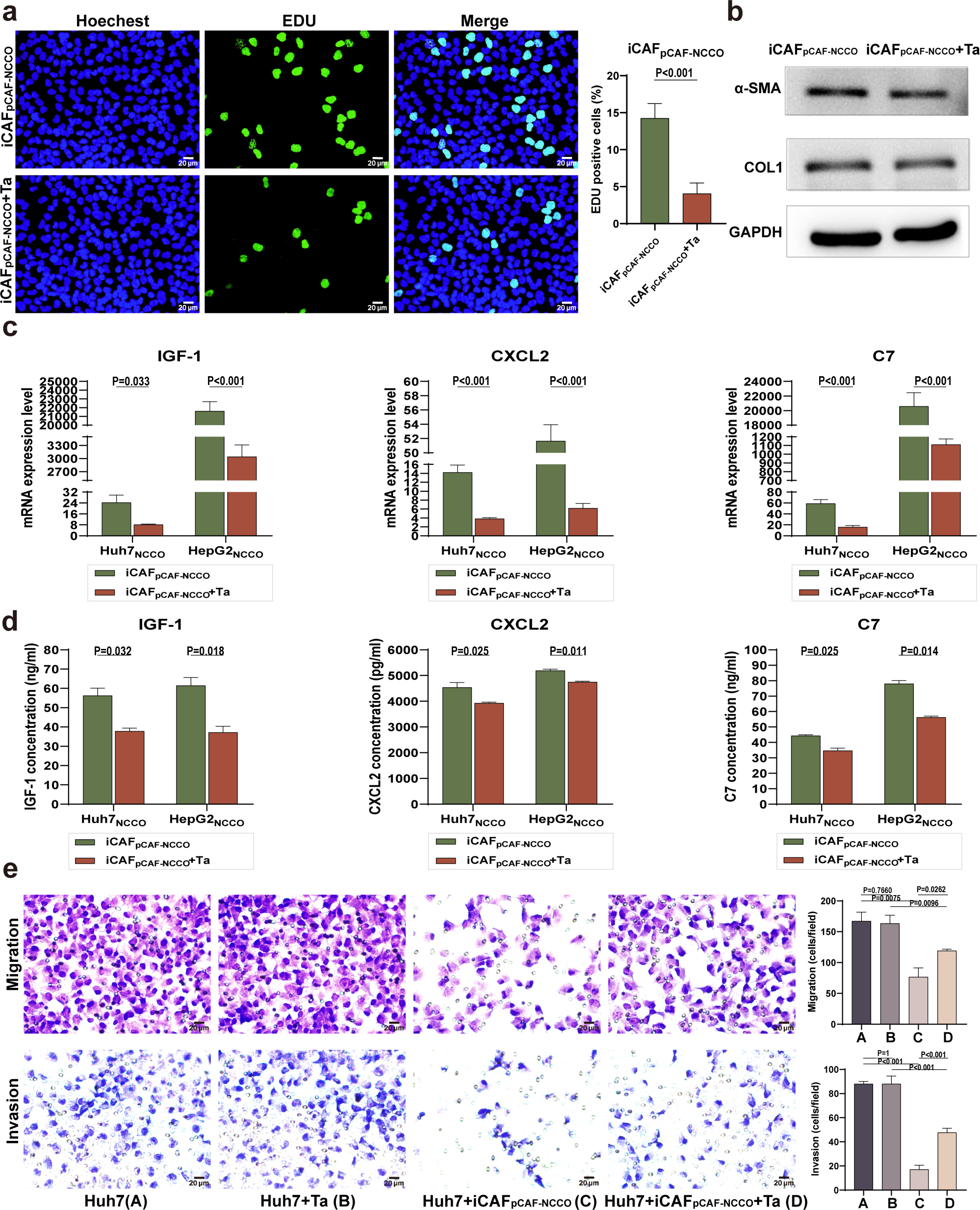

Supplement: S17 Fig — a, The impact of taurine (Ta) on iCAFpCAF-NCCO proliferation was determined using the EDU assay. b, western blot analysis of the effect of taurine on α-SMA and COL1 expression in iCAFs pCAF-NCCO. c and d, qPCR (c), and ELISA assays (d) confirmed the expression of IGF-1, CXCL2, and C7 in iCAFpCAF-NCCO after taurine treatment. e, Transwell co-culture assays examined the effect of iCAFpCAF-NCCO on tumor cells migration and invasion with or without taurine. CAF, cancer-associated fibroblast; iCAF, inflammatory cancer-associated fibroblast; pCAF, primary cancer-associated fibroblast; NCCO, non-contact co-culture; EDU, 5-ethynyl-2′-deoxyuridine; qPCR, quantitative polymerase chain reaction; IGF-1, insulin-like growth factor 1; CXCL2, C-X-C motif chemokine ligand 2; C7, complement component 7; ELISA, enzyme-linked immunosorbent assay; COL1, collagen I; α-SMA, α-smooth muscle actin; GAPDH, glyceraldehyde-3-phosphate dehydrogenase. Statistical analysis was performed using the Student t test (a, c, d and e). (TIF) [file pmed.1004703.s019.tif]
